# Aging shifts mitochondrial dynamics toward fission to promote germline stem cell loss

**DOI:** 10.1111/acel.13191

**Published:** 2020-07-14

**Authors:** Oyundari Amartuvshin, Chi‐Hung Lin, Shao‐Chun Hsu, Shih‐Han Kao, Alvin Chen, Wei‐Chun Tang, Han‐Lin Chou, Dong‐Lin Chang, Yen‐Yang Hsu, Bai‐Shiou Hsiao, Elham Rastegari, Kun‐Yang Lin, Yu‐Ting Wang, Chi‐Kuang Yao, Guang‐Chao Chen, Bi‐Chang Chen, Hwei‐Jan Hsu

**Affiliations:** ^1^ Molecular and Cell Biology Taiwan International Graduate Program Academia Sinica Taipei Taiwan; ^2^ Graduate Institute of Life Science National Defense Medical Center Taipei Taiwan; ^3^ Institute of Cellular and Organismic Biology Taipei Taiwan; ^4^ Imaging Core Facility at the Institute of Cellular and Organismic Biology Academia Sinica Taipei Taiwan; ^5^ Research Center for Applied Science Academia Sinica Taipei Taiwan; ^6^ The Affiliated Senior High School of National Taiwan Normal University Taipei Taiwan; ^7^ Institute of Biological Chemistry Academia Sinica Taipei Taiwan; ^8^ Present address: Institute of Chemistry Academia Sinica Taipei Taiwan

**Keywords:** BMP, Drp1, GSC, Marf, mitochondrial fission, mitochondrial fusion

## Abstract

Changes in mitochondrial dynamics (fusion and fission) are known to occur during stem cell differentiation; however, the role of this phenomenon in tissue aging remains unclear. Here, we report that mitochondrial dynamics are shifted toward fission during aging of *Drosophila* ovarian germline stem cells (GSCs), and this shift contributes to aging‐related GSC loss. We found that as GSCs age, mitochondrial fragmentation and expression of the mitochondrial fission regulator, Dynamin‐related protein (Drp1), are both increased, while mitochondrial membrane potential is reduced. Moreover, preventing mitochondrial fusion in GSCs results in highly fragmented depolarized mitochondria, decreased BMP stemness signaling, impaired fatty acid metabolism, and GSC loss. Conversely, forcing mitochondrial elongation promotes GSC attachment to the niche. Importantly, maintenance of aging GSCs can be enhanced by suppressing Drp1 expression to prevent mitochondrial fission or treating with rapamycin, which is known to promote autophagy via TOR inhibition. Overall, our results show that mitochondrial dynamics are altered during physiological aging, affecting stem cell homeostasis via coordinated changes in stemness signaling, niche contact, and cellular metabolism. Such effects may also be highly relevant to other stem cell types and aging‐induced tissue degeneration.

## Graphical abstract

Aging shifts mitochondrial balance toward fission; fragmented mitochondria with low membrane potential (△Ψ), and ROS levels, along with decreased BMP signaling causing GSC loss. Marf depletion induces highly fragmented mitochondria with low fatty acid (FA) oxidation, causing oil droplet (LD) accumulation, and attenuated BMP signaling that cause GSC loss. Drp1 depletion generates elongated mitochondria and increased E‐cadherin expression to strengthen GSC competitiveness for niche occupancy.

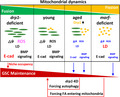

## 1 INTRODUCTION

Stem cells reside in a specialized microenvironment called the niche, which provides both physical contact and stemness factors that ensure and maintain the stem cell fate (Morrison & Spradling, 2008). While stem cells promote tissue longevity by continually producing differentiated cells, the maintenance and/or function of stem cells often decrease with age, leading to aging‐dependent tissue degeneration (Ahmed, Sheng, Wasnik, Baylink, & Lau, 2017; Schultz & Sinclair, 2016). However, the mechanisms by which aging affects stem cells are only partially understood.

Mitochondria frequently undergo coordinated cycles of fusion and fission (known as mitochondrial dynamics) to properly adjust the shape, size, and cellular distribution of the organelle to meet specific cellular requirements (Hoppins, Lackner, & Nunnari, 2007; McQuibban, Lee, Zheng, Juusola, & Freeman, 2006). Fusion produces elongated mitochondria by respectively joining the outer and inner membranes of two mitochondria. The closely related Dynamin‐related GTPases, Mitofusin (Mfn) 1 and Mfn2, mediate outer membrane fusion, while optic atrophy (Opa1) is integral for fusion of the inner membrane (Pernas & Scorrano, 2016). On the other hand, excessive mitochondrial fission produces fragmented mitochondria and is mediated by another Dynamin‐related GTPase, called Dynamin‐related protein (Drp1) (Pernas & Scorrano, 2016). Drp1 is recruited by its receptors on the outer membrane and oligomerizes along the mitochondrial constriction site to constrict the organelle and induce scission (Pernas & Scorrano, 2016). The two *Drosophila* homologues of Mfn1/2 are Fuzzy onion (Fzo) and Mitochondrial assembly regulatory factor (Marf) (Hales & Fuller, 1997; Hwa, Hiller, Fuller, & Santel, 2002). Fzo is exclusively expressed in the testes, while Marf is expressed in the germline and somatic cells (Hwa et al., 2002). *Drosophila* also has single homologues of Opa1 and Drp1, which have the same names as their mammalian counterparts (Verstreken et al., 2005; Yarosh et al., 2008).

Mitochondrial dynamics are known to influence several mitochondria‐dependent biological processes, such as lipid homeostasis, calcium homeostasis, and ATP production (Tilokani, Nagashima, Paupe, & Prudent, 2018). Recent studies have also proposed a role for mitochondrial fusion and fission in regulating stem cell fate (Fu, Liu, & Yin, 2019; Seo, Yoon, & Do, 2018). In one interesting example, murine neural stem cells were shown to exhibit elongated mitochondria, and depletion of Mfn1 or Opa1 impaired their self‐renewal (Khacho et al., 2016). Despite tantalizing observations such as these, the overall impact of mitochondrial dynamics in aging stem cells and the mechanisms by which mitochondrial dynamics might affect stem cell function remain unclear.

**FIGURE 1:**
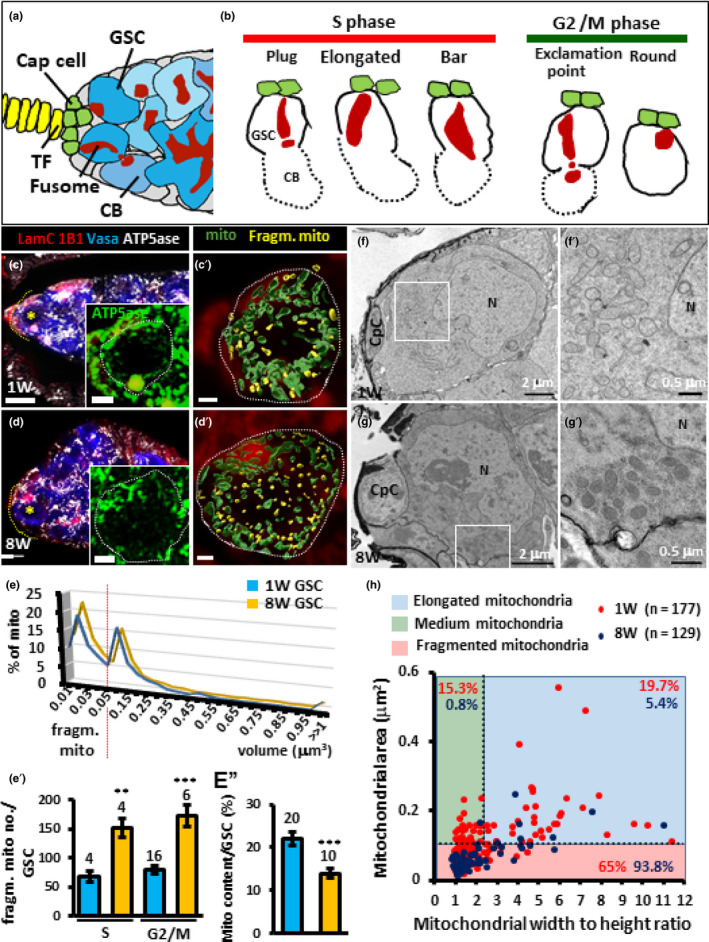
Aged GSCs exhibit increased numbers of fragmented mitochondria. (a) An illustration of the anterior part of the *Drosophila* germarium. Terminal filament (TF) and cap cells form the GSC niche to house GSCs. Each GSC carries a fusome. The cystoblast (CB), the immediate daughter cell of the GSC, undergoes four rounds of incomplete division to form a 16‐cell cyst. Each cyst carries a branched fusome that interconnects germ cells within the cyst. (b) Fusome morphology changes according to the GSC cell cycle phase. During S phase, GSCs display a “plug,” “elongated,” or “bar” fusome morphology, as a nascent fusome (or plug) is assembled and then fused to the original fusome, thereby connecting the GSC and the cystoblast. During early G2, GSCs exhibit “exclamation point” fusome morphology, as the connection between GSCs and the cystoblast is severed. During late G2 and M, GSC fusomes display a “round” fusome. (c and d) One‐ (c) and 8‐week‐old germaria (d) with LamC (red, TF and cap cell nuclear envelopes), 1B1 (red, fusomes), Vasa (blue, germ cells), and ATP5ase (gray, mitochondria). Inserts are higher magnification of GSCs marked by yellow asterisks in E and F, with ATP5ase shown in green. (c′ and d′) are same GSCs shown in the inserts, but with less layers and the surface model of mitochondria from Imaris shown. Mitochondria (mito) forming networks are shown in green; fragmented mitochondria (fragm. mito) are shown in yellow. Asterisks indicate GSC(s) in the germarium; dashed circles outline GSCs. Yellow dashed lines outline the anterior edge of the germarium. Scale bars in c and d are 5 μm, in the insert of c and d are 2 μm, and in c′ and d′ are 1 μm. (e) Percentage (%) of mitochondria with indicated volume in 1‐ and 8‐week‐old GSCs. (e′) Number (no) of fragmented mitochondria in 1‐ and 8‐week‐old GSCs at S or G2/M phases. (e″) Percentage of mitochondrial content per GSC in 1‐ and 8‐week‐day‐old GSCs. Numbers of analyzed GSCs are shown above each bar. Error bars, *SEM*. **p < 0.01; ***p < 0.001. (f and g) Representative electron micrographs of the anterior regions of 1‐ (f) and 8‐week (W)‐old germaria (g). f′ and g′ are enlarged views from the areas indicated by squares in f and g. N, Nucleus. CpC, cap cells. Scale bars in e and g are 2 μm, and bars in f′ and g′ are 0.5 μm. (h) The area (μm^2^) and the width to height (W/H) ratio of individual mitochondria in 1‐ (red) and 8‐week‐old GSCs (blue). Mitochondria distributed in blue, green, and pink areas are elongated, medium, and fragmented mitochondria, respectively. Solid and dashed lines represent the mean of W/H ratio and area of mitochondria in 1‐week‐old GSCs, respectively. n, number of analyzed mitochondria. Percentages of mitochondria in each group (1‐ versus 8‐week‐old GSCs) showed significant differences (*p *< 0.001, Chi‐squared test). Representative germaria are shown in 3D‐reconstructed images; genotype of flies is *yw*.

We used the *Drosophila* ovary to address the question of how mitochondrial dynamics affect and are affected by stem cell aging, taking advantage of the short lifespan of *Drosophila* and its amenability to powerful genetic methods. Most importantly, the *Drosophila* ovary houses well‐characterized germline stem cells (GSCs) (Figure 1a) (Kirilly, Spana, Perrimon, Padgett, & Xie, 2005), which gradually escape the niche and become differentiated during aging (Kao et al., 2015). A *Drosophila* ovary contains 16–20 egg‐producing functional units, which are called ovarioles (Spradling, 1993). The germarium is the anterior‐most structure of the ovariole, and it houses two to three GSCs at its anterior tip. The terminal filament, cap cells, and anterior escort cells are also located in the anterior tip of the germarium and form the GSC niche (Losick, Morris, Fox, & Spradling, 2011). GSCs directly contact niche cap cells (the major niche component)(Song & Xie, 2002), and each of GSC contains a fusome, an organelle with a membranous‐like structure that is juxtaposed to the GSC‐cap cell interface (Xie & Spradling, 2000). As a single asymmetric GSC division gives rise to a cystoblast (CB), the fusome changes morphology according to the stage of the cell cycle (Figure 1b). During G2/M phase, the GSC fusome is round. Then, at G1 and S phases, it grows and fuses with a newly formed fusome destined for the daughter CB, generating an elongated fusome. This elongated fusome is pinched off when the GSC and CB begin to separate during early G2 phase, leading it to regain its round shape in the GSC until the end of M phase (de Cuevas & Spradling, 1998; Kao et al., 2015). After M phase, the daughter CB undergoes four rounds of incomplete division to form a 16‐cell cyst; each germ cell within the cyst is interconnected by a branched fusome (Spradling, 1993). Next, the 16‐cell cyst is surrounded by a layer of follicle cells, and the whole structure buds off from the germarium, finally developing into a mature egg (Spradling, 1993). Mitochondria are generally found in a big cluster located near the fusome in GSCs. In contrast, highly fragmented mitochondria are located far from the fusome in 4‐ and 8‐cell cysts, while elongated mitochondria are observed in close proximity to the fusome in 16‐cell cysts (see Figure 4b) (Cox & Spradling, 2003).

In this study, we used fluorescence and transmission electron microscopy (TEM) to show that fragmented mitochondria are increased in aged GSCs in fixed ovarian tissues. Our live‐imaging data further show that mitochondrial fission is increased in aged GSCs, suggesting a source for the accumulated fragmented mitochondria. GSCs with mitochondrial fragmentation forced by a mutation of *marf* mimic aged GSCs, which divide slowly, exhibit low Dpp (BMP orthologue) stemness signaling and have a tendency to leave the niche and differentiate (Kao et al., 2015). Furthermore, the fragmented mitochondria exhibit reduced membrane potential and defective fatty acid metabolism, as revealed by cytoplasmic oil droplet accumulation. On the other hand, stimulating mitochondrial elongation in GSCs by *drp1* mutation increases GSC**–**niche occupancy, at least in part, through increased expression of E‐cadherin. Notably, cytoplasmic oil droplet accumulation is not frequently observed in aged GSCs, possibly because the aged cells have less severe mitochondria fragmentation compared to *marf* mutant GSCs. Interestingly, Drp1 expression is increased in aged GSCs, and suppressing Drp1 expression to prevent mitochondrial fission or treating the flies with rapamycin to induce autophagy reduces aging‐dependent GSC loss. Together, our results show that aging shifts mitochondrial dynamics toward fission in stem cells, and this shift impairs the maintenance of stemness factor production and cellular metabolism. Thus, mitochondrial homeostasis may be an interesting target for modulating aging‐related tissue degeneration.

## 2 RESULTS

### 2.1 Aging increases fragmented mitochondria in GSCs

To understand whether aging affects mitochondrial morphology, we first labeled germaria with antibodies for LamC, 1B1, and Vasa to delineate the various cell types. We also labeled ATP synthase 5 α subunit (ATP5ase) in the mitochondria of young (1‐week‐old) and aged ovaries (8‐week‐old) to analyze mitochondrial location and size. In young GSCs (indicated by an asterisk in Figure 1c), mitochondria formed a big cluster near the fusome, an observation that is in agreement with a previous report (Cox & Spradling, 2003); this location was not changed in aged GSCs (indicated by an asterisk in Figure 1d). The volume of individual mitochondria in GSCs was about 0.4 ± 0.14 μm^3^ (*n* = 20 GSCs) on average, but the size ranged up to 48 μm^3^ (data not shown). Notably, the sizes of individual mitochondria and numbers of mitochondria in a cluster could not be accurately assessed due to limitations in resolution. However, we could find that mitochondria with sizes smaller than 0.05 μm^3^ were increased in aged GSCs compared to young GSCs (Figure 1e,e′, and see yellow signals in Figure 1c′,d′); we defined these small mitochondria as fragmented mitochondria. This difference was not correlated with the phase of the GSC cell cycle (Figure 1e′). In addition, total mitochondrial content (ratio of total mitochondrial volume to the GSC volume) was significantly lower in aged GSCs than in young GSCs (Figure 1e″). The immediate daughter cells of GSCs (CBs) also displayed a wide range of mitochondrial sizes, as well as age‐associated increases in fragmented mitochondria and decreases in mitochondrial content (Figure S1A‐C), indicating that GSCs and CBs share similar responses of mitochondrial characteristics to aging.

Next, we used TEM to further examine mitochondria in GSCs of young (1‐week‐old) and aged ovaries (8‐week‐old) at high resolution (Figure 1f,g,f′,g′). GSCs were identified by their anterior location in the germarium and direct contact with cap cells; we measured mitochondrial areas and width to height (W/H) ratios only in GSCs where the nucleus could be clearly identified. Despite the fact that organelle orientation in the thin section will affect the size measurement, we reasoned that the stochastic nature of mitochondrial orientation should still allow us to see a difference between experimental groups if mitochondria were more fragmented in aged GSCs. Indeed, averages of mitochondrial area and length were larger in young GSCs (area: 0.11 ± 0.11 μm^2^; W/H ratio: 2.34 ± 1.9, 177 mitochondria from 6 GSCs), as compared to aged GSCs (area: 0.06 ± 0.04 μm^2^, *p *< 0.05; W/H ratio: 1.92 ± 1.4, *p *< 0.001, 129 mitochondria from 5 GSCs) (Figure 1h). For further analysis, mitochondria were classified into three groups according to the mean area and W/H (of mitochondria analyzed in young GSCs: mitochondria with areas bigger than 0.11 μm^2^ and W/H ratio larger than 2.34 were considered “elongated mitochondria,” mitochondria with areas bigger than 0.11 μm^2^ and W/H ratio smaller than 2.34 were counted as “medium mitochondria,” and mitochondria with areas smaller than 0.11 μm^2^ were called “fragmented mitochondria” (Figure 1h). The respective percentages of elongated, medium, and fragmented mitochondria in young GSCs were 19.7%, 15.3%, and 65% versus 5.4%, 0.8%, and 93.8% (*p *< 0.001) in aged GSCs (Figure 1h). Thus, we found that aged GSCs exhibited high levels of fragmented mitochondria, consistent with our conclusion from fluorescence microcopy experiments. Because the mitochondria in aged GSCs were smaller than those in young cells, we suspected that mitochondria in aged cells may either undergo less fusion or more fission to yield fragmented mitochondria.

### 2.2 Aged GSCs display a preference for mitochondrial fission

**FIGURE 2:**
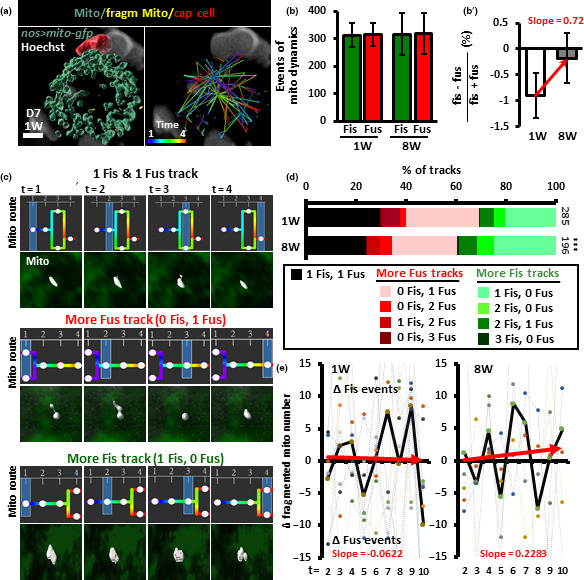
Mitochondrial dynamics favor fission in aged GSCs. (a) Left panel: Mito‐GFP‐labeled mitochondria (blue) in 1‐week (W)‐old GSCs with Hoechst labeling (gray) were analyzed at the first 4 time points and displayed as a surface‐rendered 3D trace. The cap cells were recognized by their small ovoid nucleus shown in red and juxtaposition with GSCs. Fragmented mitochondria are shown in yellow. Right panel: tracking route of mitochondria in the GSC. Color code indicates mitochondrial route from time point 1 to 4. Scale bar, 2 μm. (b) Mitochondrial dynamics events [fission (fiss) plus fusion (fus)] of 1‐ and 8‐week‐old GSCs within the first 10 time points. (b′) Proportional difference of fission and fusion in 1‐ and 8‐week‐old GSCs. ns, no significance. (c) Representative tracks of mitochondria (shown in gray) analyzed over 4 time points are classified into three groups: mitochondrion undergoing 1 fusion and 1 fission (top panels), more fission (middle panels, only one fission track is shown as an example), and more fusion (bottom panels, only one fusion track is shown as an example). Mitochondrial routes are indicated by the color code shown in the right panel of a. White circles represent tracked mitochondrion, and blue squares indicate the analyzed time point. (d) Percentage of mitochondrial tracks of each type in 1‐ and 8‐week‐old GSCs analyzed throughout time points 1‐4, 4‐7, and 7‐10. Track numbers analyzed are shown beside each bar. ***p < 0.001, chi‐squared test. (e) Net difference (△) of fragmented mitochondrial number between two time points within the first 10 recorded from 1‐ and 8‐week GSCs. Positive and negative values indicate net preference for fission and fusion events, respectively. Dashed lines show fluctuation of fragmented mitochondrial number for each GSC; solid lines show the average fluctuation of fragmented mitochondrial number. Red solid lines shown in b′, and e are trend‐lines; slopes represent tendencies toward fusion or fission.

To distinguish between the two possibilities described above, we first labeled mitochondria in live young (1‐week‐old) and aged ovaries (7‐week‐old) by MitoTracker, a fluorescent dye (Figure S1D and E). We found that mitochondria in aged GSCs were more fragmented than those in young GSCs (insets in Figure 1c,d); however, we sometimes could not distinguish if analyzed mitochondria were from GSCs or somatic cells. We, therefore, expressed *mito*‐*gfp* [a marker for mitochondria (Cox & Spradling, 2003)] in the germline using *nos*‐*GAL4*. We made live recordings of *nos*>*mito*‐*gfp* GSCs for 10 min (300 time points with intervals of about 2 s to generate 300 stacks, each stack containing 100 slices along the z‐axis) using lattice light‐sheet microscopy (Movie S1 and Movie S2), which allows us to capture images with high resolution and fast acquisition speed (Chen et al., 2014). Hoechst staining was used to identify cap cells according to their small oval‐shaped nuclei and anterior‐most location in the germaria; GSCs were identified by their direct contact with cap cells (left panel in Figure 2a). We found that over the 10‐min recording period, the mitochondria number nearly doubled (1 week‐old, 9 GSCs: 0 min, 69 ± 21 mitochondria versus 10 min, 139 ± 17 mitochondria; 8 week‐old, 5 GSCs: 0 min, 189 ± 45 mitochondria versus 10 min, 346 ± 95 mitochondria) (Figure S2A,B). We suspected that this result might be due to noisy signal as a result of Mito‐GFP photobleaching, or the laser itself may induce mitochondrial fission. Given that mitochondrial number should not dramatically change under normal physiological conditions, we limited our analysis to the first 10 stacks; during this period, the mitochondrial number was steadily maintained (Figure 2c,d). In this experiment, we noted that aged GSCs exhibited decreased mitochondrial content and an increased proportion of fragmented mitochondria (mitochondrial size <0.05 μm^3^, as yellow mitochondria in the left panel of Figure 2a), consistent with our other results.

To examine mitochondrial dynamics, we carefully tracked mitochondrial fission and fusion over three periods of four time points each, covering the first ten live images (i.e., time points 1‐4, 4‐7, and 7‐10) of young and aged GSCs (Figure 2a, right panel and Movie S3 show representative tracking routes from time points 1‐4). If a fission or fusion event occurred on a single track in each of the three time periods, the maximum total fusion‐plus‐fission events would be 3 for each track, allowing us to make a simple characterization of mitochondrial dynamics. We found that total fission and fusion events in young and aged GSCs were similar (Figure 2b; see examples of fission and fusion in Figure 2c). However, the proportional difference between fission and fusion was slightly more in aged GSCs (Figure 2b′), suggesting that there may be a minor excess of fission causing a very slow accumulation of fragmented mitochondria during aging. To further analyze the mitochondria tracking data, we grouped tracks into three types (Figure 2c): tracks with one fusion and one fission event (one fis and fus track, top panel), tracks with higher number of fission events than fusion events (fis > fus track, middle panel), and tracks with lower number of fission events than fusion events (fis < fus track, bottom panel). Our results showed that the respective proportions of one fiss and fus, fiss > fus and fiss < fus tracks were 30%, 31%, and 40% (*n* = 285) in young GSCs and 24%, 40%, and 36% in aged GSCs (*n* = 196, *p *< 0.001). These results showed that one‐third of mitochondrial dynamic events were balanced with equal amounts of fusion and fission in young GSCs, while these balanced tracks were decreased in aged flies (Black bar in Figure 2d). Moreover, mitochondria preferred to fuse with other mitochondria in young GSCs, while more mitochondria were observed undergoing fission in aged GSCs (Figure 2d). These results indicate that young GSCs display a slight preference for fusion, while aged GSCs show a small preference for fission.

To confirm these results, we also counted the numbers of fragmented mitochondria at each time point, as any change in their number should reflect the relative proportions of fission and fusion events. For example, if one fragmented mitochondrion is present at the first time point but two fragmented mitochondria are present at the next time point (the resulting change in number of fragmented mitochondria is +1), the cell should exhibit a net tendency toward fission. In contrast, if two fragmented mitochondria are present at the first time point but only one is present at the next time point (the resulting change in number of fragmented mitochondria is −1), the cell should exhibit a net tendency toward fusion. We averaged the changes in numbers of fragmented mitochondria between stacks for all GSCs and found that the trendline of net changes of mitochondrial dynamics in young GSCs was sloped slightly toward fusion, while in aged GSCs, the trendline sloped toward fission (Figure 2e). Of note, the changes in mitochondrial number varied greatly (both positive and negative) in both young and aged GSCs, implying mitochondrial fission and fusion events are highly frequent in GSCs. Taken together, our live‐imaging data show that aging of GSCs prompts a slight shift in the balance of mitochondrial dynamics toward fission.

### 2.3 Preventing mitochondrial fusion decreases GSC division and maintenance

**FIGURE 3:**
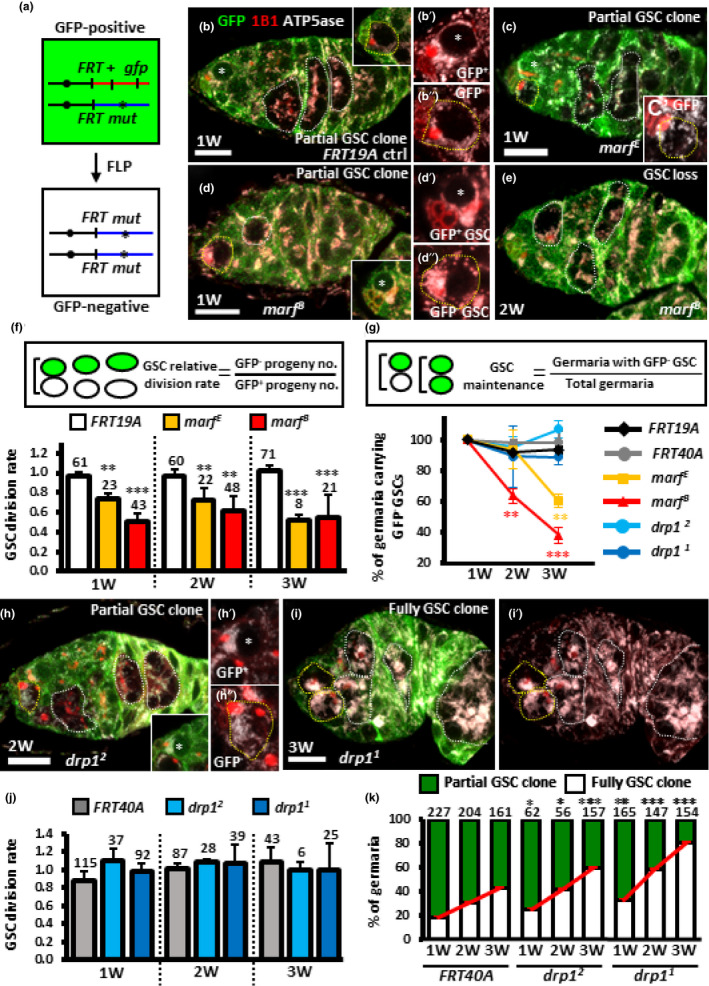
Disrupting mitochondrial fusion decreases GSC division and maintenance while disrupting mitochondrial fission promotes GSC–niche occupancy. (a) Mitotic recombination was used to generate GSC mutants for *marf* or *drp1*. Females carrying a wild‐type (wt, +) allele linked to a marker gene (*gfp*) *in trans* with a mutant (*mut*) allele were generated. FLP‐mediated recombination between FRT sites during mitotic division generates a homozygous mutant cell, identifiable by the absence of marker expression. (b‐e, h and i) *FRT19A* control (ctrl) (b), *marf^E^* (c), *marf^B^* mutant mosaic germaria (d and e), *drp1^2^* (h) and *drp1^1^* mutant mosaic germaria (i) with GFP (green, wt cells), 1B1 (red, fusomes), and ATP5ase (gray, mitochondria) at 1 (b‐d), 2 (e and f), and 3 weeks (w) after clone induction (ACI) (i). Wt GSCs are indicated by asterisks; GFP‐negative GSCs and their daughter cells are outlined by yellow and white dashed lines, respectively. Inserts in b and c show GFP‐negative (−) GSCs, in d and h show GFP‐positive (+) GSCs at different focal planes. b′ and b″, c′, d′, d″, h′ and h″ show higher magnifications of GFP^+^ or GFP^−^ GSCs with 1B1 and ATP5ase staining. In the germarium (d), GFP^−^ GSCs only produce one GFP^−^ GSC daughter cell, indicating a low rate of GSC division; the germarium (e) carries GFP^−^ germ cells but not GFP^−^ GSCs, indicating the loss of GFP^−^ GSCs. Germaria carrying GSCs that are not all GFP‐negative are referred to as partial GSC clones, while germaria carrying GSCs that are all GFP‐negative are referred to as full GSC clones. Scale bar, 10 µm. (f and j) GSC relative division rates (ratio of GFP‐negative to GFP‐positive GSC progeny) in mosaic germaria. The number of GSCs analyzed is shown above each bar. (g) Relative percentage of GSC clones (as a proportion of total GSCs) at 1, 2, and 3 W ACI. (k) Relative percentage of germaria with partial GSC clones versus germaria with full GSC clones at 1, 2, and 3 W ACI. Numbers of germaria analyzed are shown above each bar. *p < 0.05; **p < 0.01; ***p < 0.001. Error bars, mean ± *SEM*.

GSC division and maintenance are reduced during aging (Kao et al., 2015; Pan et al., 2007; Zhao, Xuan, Li, & Xi, 2008). To understand whether mitochondrial fission is involved in this process, we introduced a mutation in the fusion regulator, *marf*, in GSCs by mitotic recombination (indicated by the absence of GFP) (Figure 3a–e). As expected, *marf* mutant GSCs displayed fragmented mitochondria (Figure 3c′,d″) when compared to neighboring control GSCs or the GSCs in control germaria (Figure 3b′,b″,d′). We then assessed whether preventing fusion decreases GSC division (Figure 3f). Because each CB or cyst is derived from one GSC division, the ratio of mutant (GFP‐negative, GFP^−^) and control progeny (GFP‐positive, GFP^+^) can reflect relative rates of division (LaFever & Drummond‐Barbosa, 2005). We counted the number of control and mutant cystoblasts and cysts in *marf* germaria containing at least one control and one mutant GSC. The relative numbers of wild‐type and mutant CBs or cysts were unaffected by early germline death (Figure S4), and the numbers of progeny derived from control GSCs were approximately equal to those without GFP in mock mosaic germaria (relative division rate equal to approximately 1.0) at 1, 2, and 3 weeks after clone induction (ACI) (Figure 3f). In contrast, division was significantly reduced in GSCs homozygous for *marf^E^*, a hypomorphic allele, and for *marf^B^*, a null allele (Yarosh et al., 2008) (Figure 3f). We next asked whether diminished fusion reduces GSC maintenance by counting the number of germaria carrying *marf* mutant GSCs over time (Figure 3g and Table S1). At 3 weeks ACI, about 93 ± 4% of *FRT19A* control germaria (*n* = 336) retained at least one GFP‐negative control GSC from the first week, indicating that up to ~7% of GSCs may be naturally turned over. In 3‐week ACI mutant germaria, only 68 ± 15% (*marf^E^*, *n* = 251) and 38 ± 9% (*marf^B^*, *n* = 310) of mutant GSCs were maintained. Since we did not detect any apoptotic *marf* mutant GSCs (Figure S4), we suspect that *marf* mutant GSCs leave the niche and undergo differentiation. These results show that mitochondrial fragmentation is detrimental to GSC division and maintenance.

### 2.4 Forcing mitochondrial elongation promotes GSC competitiveness for niche occupancy

**FIGURE 4:**
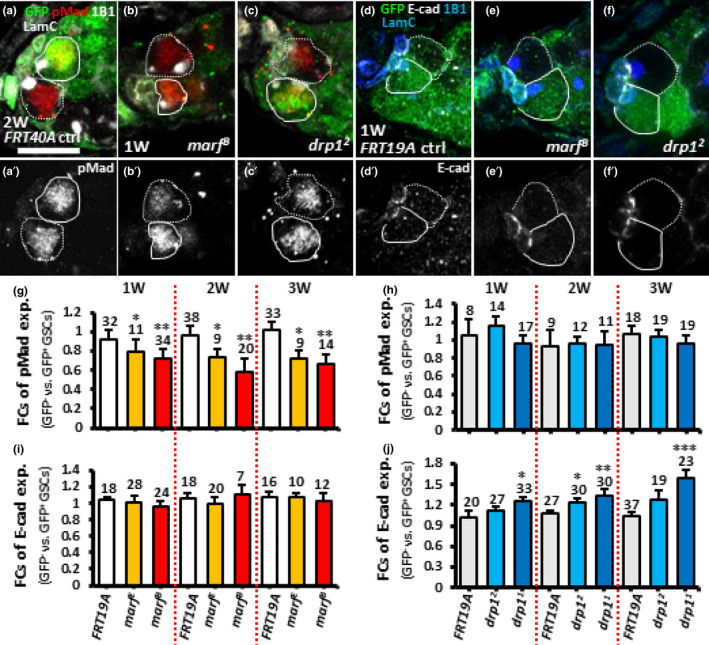
Disrupting mitochondrial fusion in GSCs decreases BMP signaling, while disrupting mitochondrial fission promotes E‐cadherin expression in the GSC‐cap cell junction. (a–c) *FRT40A* control (ctrl) (a), *marf^B^* (b) and *drp1^2^* mosaic germaria (c) with GFP (green, wild‐type cells), 1B1 (gray, fusomes), LamC (gray, terminal filament [TF] and cap cell nuclear envelopes), pMad (red, BMP signaling) 1 week (W) (b and c) and 2 weeks (a) after clonal induction (ACI). (d‐f) *FRT 19A* control (d), *marf^B^* (e) and *drp1^2^* mosaic germaria (f) with GFP (green, wild‐type cells), 1B1 (blue), LamC (blue), E‐cad (gray) 1‐week ACI. (g–j) Fold changes (FCs) of pMad (g and h) and E‐cad expression (i and j) in GFP^−^ vs GFP^+^ GSCs in the germaria with indicated genotypes 1, 2, and 3 W ACI. Numbers of analyzed GSCs are shown above each bar. *p < 0.05; **p < 0.01; ***p < 0.001. Error bars, mean ± *SEM*. Scale bar, 10 μm.

We also examined the impact of promoting elongated mitochondria on GSC homeostasis by generating GSCs with a mutation of mitochondrial fission regulator, *drp1* (indicated by the absence of GFP). GSCs homozygous for *drp1^1^* or *drp1^2^* null alleles (Yarosh et al., 2008) displayed large mitochondrial clusters that occupied a major portion of the cell (Figure 3h,h′,h″,i,i′). However, *drp1* mutant GSCs exhibited comparable division and maintenance rates compared to control GSCs (Figure 3g,j). These results indicate that preventing mitochondrial fission neither decreases GSC division nor maintenance. Interestingly, we found that the proportions of mutant germaria carrying at least one GFP‐positive GSC (partial GSC clone) decreased from 75% to 34% (*drp1^2^*) and 70% to 14% (*drp1^1^*), while the proportion of germaria in which all GSCs were mutant (full GSC clone) increased from 25% to 66% (*drp1^2^*, 41% increase) and 30% to 86% (*drp1^1^*, 56% increase) by 3‐week ACI (Figure 4k). In *FRT40A* mock mosaic germaria, only a 25% increase was observed, due to the natural loss of neighboring GFP‐positive GSCs (Figure 3k). These results indicate that GSCs with *drp1* mutation (shifting mitochondrial dynamics balance toward fusion) tend to push away neighboring control GSCs to dominate niche occupancy.

### 2.5 Marf promotes Dpp stemness signaling while Drp1 suppresses GSC–niche attachment

We next investigated whether regulation of GSC maintenance by mitochondrial dynamics relies on Dpp stemness signaling (Xie & Spradling, 1998) or E‐cadherin‐mediated GSC**–**niche adhesion (Song & Xie, 2002); both are known to be decreased during aging (Pan et al., 2007; Tseng et al., 2014). We found that in mock mosaic germaria, expression levels of phosphorylated Mad (pMad) revealed Dpp signaling (Song et al., 2004) was similar in GFP‐negative and GFP‐positive GSCs at 1‐, 2‐, and 3‐week ACI (Figure 4a,g). However, pMad expression was significantly reduced in *marf^E^* or *marf^B^* mutant GSCs compared to neighboring GFP‐positive control GSCs (Figure 4b,g). The level of pMad remained similar in *drp1* mutant GSCs (Figure 4c,h) and control GSCs over time. On the other hand, E‐cadherin expression was similar in GFP‐positive and GFP‐negative GSC niches of control and *marf* mutant germaria (Figure 4d,e,i). Meanwhile, E‐cadherin expression was significantly increased in GSC**–**niche junctions of *drp1^1^ or drp1^2^* mutant germaria as compared to neighboring normal GSC**–**niche junctions at 1‐, 2‐, and 3‐week ACI (Figure 4f,j). Moreover, stronger expression of E‐cadherin in *drp1^1^* mutant GSC**–**niche junctions compared to *drp1^2^* mutant GSC**–**niche junctions may reflect a stronger competiveness of *drp1^1^* mutant GSCs for niche occupancy (see Figure 3k). Together, these results indicate that cells with fragmented mitochondria have decreased Dpp stemness signaling in GSCs, while those with elongated mitochondria have enhanced GSC**–**niche attachment.

### 2.6 Impaired mitochondrial fusion reduces egg laying

**FIGURE 5:**
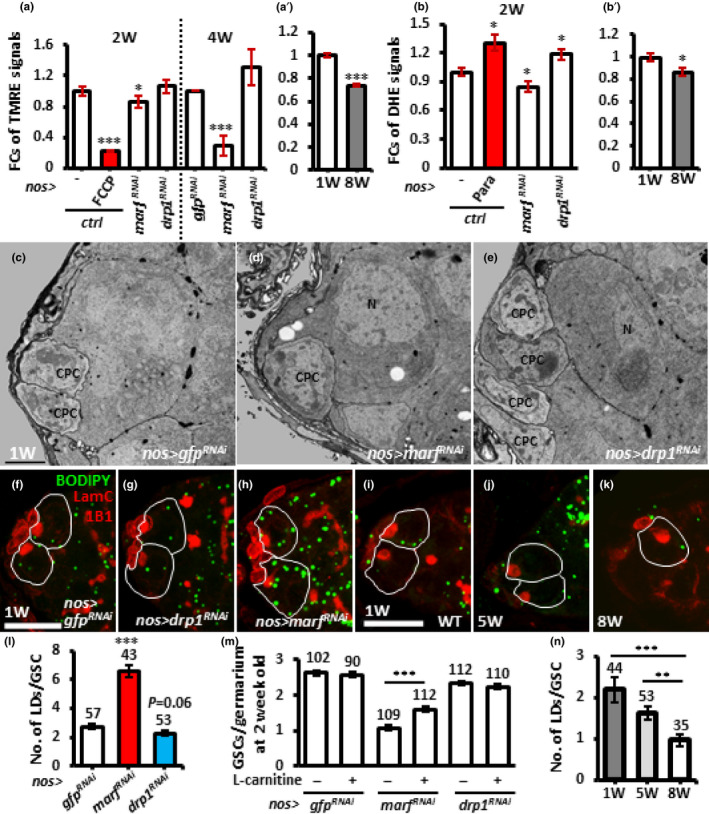
*marf*‐*knockdown* and aged GSCs display unhealthy mitochondria and reduced cellular ROS levels, while only *marf*‐knockdown GSCs exhibit oil accumulation. (a) Fold changes (FCs) of TMRE signals in isolated *bam^1^*/*bam^△86^* GSCs with indicated genotypes at 2 and 4 weeks after eclosion with or without FCCP treatment. (a′) FCs of TMRE signals in isolated *bam^1^*/*bam^△86^* GSCs of 1‐ and 8‐week‐old flies. (b) FCs of DHE signals in isolated *bam^1^*/*bam^△86^* GSCs with indicated genotypes 2 weeks after eclosion with or without paraquat treatment. (b′) FCs of DHE signals in isolated *bam^1^*/*bam^△86^* GSCs of 1‐ and 8‐week‐old flies. Each experiment was repeated 6 times, except TMRE analysis at 4 weeks was repeated 4 times. (c–e) Representative electron micrographs showing the anterior regions of 1‐week (w)‐old *nos *>* gfp^RNAi^* (c), *nos *>* marf^RNAi^* (d), and *nos *>* drp1^RNAi^* germaria (e). GSCs are identified as the large cells directly contacting cap cells (CpCs). N, nucleus. Asterisks indicate oil droplets. Scale bar, 1 μm. (f‐l) One‐week (w)‐old *nos *>* gfp^RNAi^* (f) *nos *>* marf^RNAi^* (g), *nos *>* drp1^RNAi^* (h), wild‐type (WT) (i), 5‐ (j) and 8‐week‐old germaria (k) stained for LamC (red, cap cell nuclei), 1B1 (red, fusomes), and BODIPY (green, natural oil). GSCs are outlined with solid lines. Scale bar, 10 μm. (l) Number (no.) of lipid droplets (LDs) in 1‐week‐old GSCs with indicated genotypes with or without L‐Carnitine treatment. (m) Average GSCs per germarium of 1‐week‐old flies with indicated genotypes with or without treated L‐Carnitine treatment. (n) Number of lipid droplets in 1‐, 5‐ and 8‐week‐old wild‐type (WT) GSCs. Numbers of analyzed GSCs are shown above each bar. *p < 0.05; **p < 0.01; ***p < 0.001. Error bars, mean ± *SEM*. Scale bar, 10 μm.

To determine whether mitochondrial dynamics affect egg production, we also knocked down *marf* and *drp1* specifically in the adult germline. To this end, we used *nos*‐*GAL4* and cultured flies at 18°C before eclosion then switched the flies to 29°C after eclosion. One‐week‐old *nos*>*marf^RNAi^* were smaller than *nos*>*drp1^RNAi^* (control) and *nos*>*drp1^RNAi^* ovaries (Figure S5A–C). Compared to control GSCs, *nos*>*marf^RNAi^* GSCs had highly fragmented mitochondria, while mitochondria were elongated in *nos*>*drp1^RNAi^* GSCs (Figure S5D–E), indicating the *RNAi* lines we used could recapitulate the mutant phenotypes. Consistently, *nos*>*marf^RNAi^* GSCs were more quickly lost from the germaria with age than the controls (Figure S5G). Although our clonal analysis showed that Drp1 is not required for GSC maintenance (see Figure 3), 3‐week‐old *nos*>*drp1^RNAi^* GSCs were lost faster than control GSCs (Figure 5g), possibly because varied expression of *nos*‐*GAL4* among aged GSCs in the niche created a competitive environment (Tseng et al., 2014). As a consequence, *nos*>*marf^RNAi^* ovaries had a dramatic reduction of egg production compared to *nos*>*drp1^RNAi^* and control ovaries (Figure 5h). Thus, oogenesis appears to be disturbed when mitochondria are fragmented in the germline.

### 2.7 Fragmented mitochondria in GSCs display low membrane potential and disrupted lipid homeostasis

**FIGURE 6:**
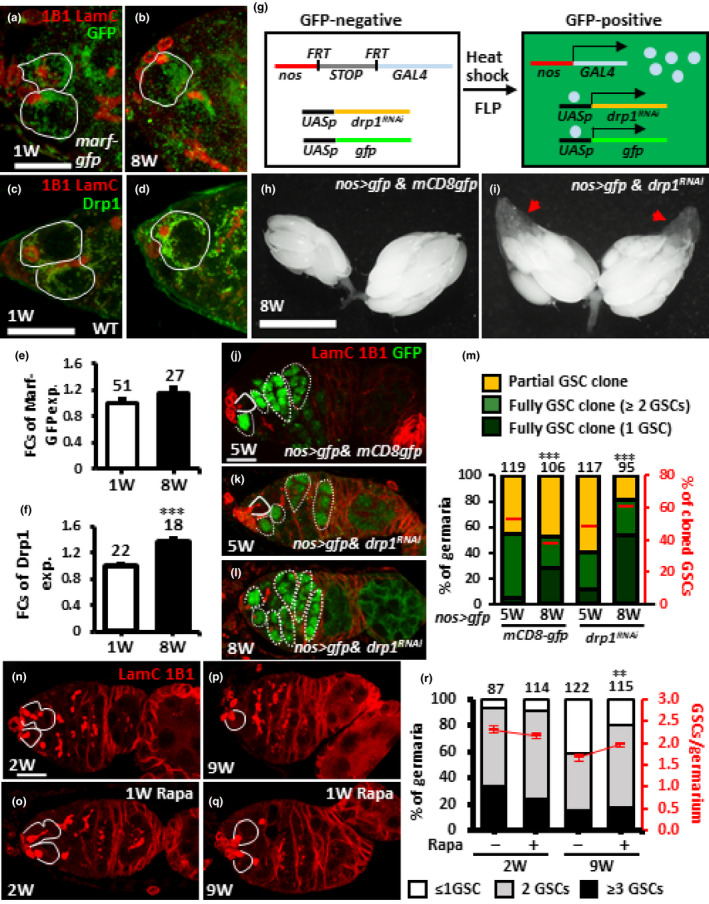
Suppression of Drp1 expression or promotion of autophagy in aged GSCs delays GSC removal from the niche. (a and b) One‐ (a) and 8‐week (W)‐old germaria (b) bearing *marf*‐*gfp* with 1B1 (red, fusomes), LamC (red, cap cell nuclear envelopes), and GFP (green, Marf) labeling. (c and d) One‐ (c) and 8‐week‐old wild‐type (WT) germaria (d) with 1B1 (red), LamC (red) and GFP (green, Drp1) labeling. (e and f) Fold changes (FCs) of Marf‐GFP (e) in *marf*‐*gfp* flies and Drp1 (f) in wild‐type flies at 1 and 8 weeks old. (g) A flip‐out system was used for *nos*‐*gal4*‐driven *drp1* knockdown in aged GSCs; knockdowns are identified by the presence of GFP expression. In females carrying a *nos* promoter‐driven FRT‐flanked flip‐in GAL4/VP16 construct (*nos *>* STOP *>* GAL4*), GAL4 is not expressed, preventing expression of *UAS*‐*gfp* and *UAS*‐*drp1^RNAi^*. GAL4 expression is turned on by removing the stop cassette through Flippase‐mediated recombination, which in turn activates expression of *UAS*‐*gfp* and *UAS*‐*drp1^RNAi^*. (g and i) 8‐week‐old *nos*>*gfp &mCD8gfp* (g) and *nos*>*gfp & drp1^RNAi^* mosaic ovaries (i) were heat‐shocked at 4 weeks old for 3 days to activate *nos*‐*GAL4*. Red arrows point to previtellogenic egg chambers. (j–l) 5‐week‐old *nos*>*gfp &mCD8gfp* (i), *nos*>*gfp and drp1^RNAi^* (j) and 8‐week‐old *nos*>*gfp and drp1^RNAi^* mosaic germaria (l) heat‐shocked at 4 weeks old for 3 days with LamC (red), 1B1 (red), and GFP (green, cloned cells) labeling. (m) Percentage (%) of 5‐ or 8‐week‐old germaria (Left y‐axis) carrying partial or full GSC clones (containing 1 or ≥2 GSCs) from flies with indicated genotypes heat‐shocked at 4 weeks old. Right *y*‐axis shows percentage of GFP‐positive GSCs in flies with indicated genotypes. (n–q) Two‐ and 9‐week‐old germaria with or without rapamycin treatment for 1 week labeled with LamC (red) and 1B1 (red). (r) Percentage of germaria with indicated GSC number of 2‐ and 9‐week‐old flies fed with or without rapamycin, beginning at 1 and 8 weeks after eclosion, respectively. Solid lines outline GSCs and dashed lines outline GSC progeny. Numbers of analyzed GSCs are shown above each bar. *p < 0.05; **p < 0.01; ***p < 0.001. Error bars, mean ± *SEM*. Scale bars in a, c, j and n are 10 μm; bar in h is 0.5 mm.

The mitochondrial membrane potential results from a proton gradient across the inner membrane, which is generated by oxidative phosphorylation complexes and is thought to reflect mitochondrial functional output. To know whether altered mitochondrial dynamics affects mitochondrial membrane potential, we used the probe, TMRE (Perry, Norman, Barbieri, Brown, & Gelbard, 2011), in isolated GSCs and analyzed TMRE signals by flow cytometry. GSCs were isolated from the germaria with *nos*‐*GAL4* driven *gfp^RNAi^*,*marf^RNAi^* or *drp1* ^RNAi^ along with *vasa*‐*gfp* (for GSC isolation) and a mutation of *bag of marbles* (*bam*, encodes a master differentiation factor) to increase GSC number (Kao et al., 2015). Isolated 2‐week‐old *gfp^RNAi^*‐knockdown (KD) GSCs were treated with FCCP, a potent mitochondrial oxidative phosphorylation uncoupler, which served as a positive control (Heytler, 1979). These FCCP‐treated GSCs showed dramatically reduced TMRE signals (Figure 5a). Compared to *nos*>*gfp^RNAi^* control GSCs, *drp1*‐KD GSCs exhibited similar levels of TMRE signal, while 2‐week‐old and 4‐week‐old *marf*‐KD GSCs displayed 19% and 71% reductions of TMRE signal, respectively (Figure 6a). This result suggests that mitochondria with impaired fusion are less functional. Furthermore, cellular ROS levels, as detected by DHE (Benov, Sztejnberg, & Fridovich, 1998), in 2‐week‐old *marf*‐KD GSCs were reduced by 16% (Figure 5b), suggesting that oxidative phosphorylation activity in fusion‐defective mitochondria is attenuated. To our surprise, like *gfp*‐KD GSCs treated with paraquat to increase ROS (Ali, Jain, Abdulla, & Athar, 1996), *drp1*‐KD GSCs displayed slightly increased ROS levels (Figure 5b), indicating that oxidative phosphorylation activity is promoted in fission‐defective mitochondria. Similar reductions of membrane potential and cellular ROS were also observed in aged GSCs (8‐week‐old), as compared to young GSCs (1‐week‐old) (Figure 5a′,b′), in line with our observation that aged GSCs carry more fragmented mitochondria.

To further explore the dysfunction of fragmented mitochondria, we also examined lipid accumulation in *marf*‐KD GSCs by TEM and a lipophilic dye (BODIPY 493/503 staining), as fatty acid oxidation takes place in mitochondria (Pakhomov et al., 2017). We observed a clear increase in number and size of oil droplets in *marf*‐KD GSCs as compared to control or *drp1*‐KD GSCs one and 2 weeks after eclosion (Figure 5c–h,l, and Figure S6), similar to previous observations in male GSCs (Sênos Demarco, Uyemura, D’Alterio, & Jones, 2019). Interestingly, promoting lipid reentry into mitochondria of 1‐week‐old *marf*‐KD GSCs, by feeding flies with L‐carnitine for 1 week (Longo, Frigeni, & Pasquali, 2016), eliminated lipid accumulation and partially rescued GSC number (Figure 5m, and Figure S6). These observations were also in agreement with a previous report that lipid homeostasis controls *Drosophila* male GSC maintenance (Sênos Demarco et al., 2019). However, we did not observe oil accumulation in aged GSCs (Figure 5i–k), possibly because mitochondrial fragmentation is less severe in aged GSCs than *marf* mutant GSCs. Thus, the nature of mitochondrial dysfunction caused by excessive fragmentation during aging appears to be complex and multifaceted, and it is unlikely that lipid accumulation alone can explain the loss of GSCs during aging.

### 2.8 Reduced fragmented mitochondria promotes maintenance of aged GSCs

Because we saw that the balance of mitochondrial dynamics in aged GSCs shifts toward fission, we further asked whether this switch is associated with changes in Marf or Drp1 expression levels. We found that according to expression of a genomic construct, *marf*‐*gfp* (Zhang, Mishra, Hay, Chan, & Guo, 2017), Marf levels in young and aged GSCs were comparable (Figure 6a,b,e). However, Drp1 expression was significantly increased in aged GSCs and their progeny, as compared to young GSCs (Figure 6c,d,f), suggesting a role for increased Drp1 in aging‐induced mitochondrial fragmentation.

To test this idea, we used the flip‐out system, in which a transcriptional stop sequence flanked by two *FRT* sites was inserted between the *nanos* (*nos*) promoter and *GAL4* (*nos*>*STOP*>*GAL4*; Figure 6g) (Ma et al., 2014). We used heat shock to express *drp1^RNAi^* or *mCD8*‐*gfp* (control) expression along with a GFP reporter specifically in germ cells of 4‐week‐old females. Strikingly, *drp1*‐KD mosaic ovaries of 8‐week‐old flies looked younger than *mCD8*‐*gfp*‐expressing ovaries in flies at the same age (Figure 6h,i), as they carried many vitellogenic egg chambers (arrow heads in Figure 6i). In addition, consistent to our previous results (see Figure 3h,i), mitochondria in *drp1*‐*KD* GSCs formed a big cluster as compared to control GSCs of 8‐week‐old flies (Figure S7), indicating a disruption of mitochondrial fission. Five‐ and 8‐week‐old *mCD8*‐*gfp*‐expressing mosaic germaria carried 52% and 38% GFP‐positive GSCs, respectively (Figure 6j,k,m), indicating that 14% of GSCs were naturally lost. However, 5‐ and 8‐week‐old *drp1*‐KD mosaic germaria, respectively, carried 49% and 61% GFP‐positive GSCs (Figure 6l,m), showing a net increase of *drp*1‐KD GSCs. In fact, 19% of 8‐week‐old *drp1*‐KD mosaic germaria carried at least one GFP‐positive GSC (partial GSC clone) and 81% carried only GFP‐positive GSCs (full GSC clone) (Figure 6m). In contrast, 59% of 8‐week‐old *mCD8gfp*‐expressing mosaic germaria were partial clones, and 41% were full GSC clones (Figure 6m). This result is consistent with the result of our clonal analysis showing that *drp1*‐deficient GSCs tend to occupy the niche. Based on these results, we conclude that decreasing *drp1* expression in aged GSCs prevents their loss.

Autophagy is suppressed by Target of rapamycin (TOR) (Kim, Kundu, Viollet, & Guan, 2011) and provides a mechanism to clear small and unhealthy mitochondria from the cell (Morita et al., 2015). To stimulate removal of fragmented mitochondria in aged flies, we fed aged flies for 1 week with rapamycin to suppress TOR. Two‐week‐old flies treated with or without rapamycin showed similar numbers of GSCs (Figure 6n,o,r), while 9‐week‐old flies treated with rapamycin showed significantly higher GSC numbers, compared to flies at the same age without rapamycin feeding (Figure 6p,q,r). These results were reminiscent of previous studies that showed autophagy promotes stem cell fate (Boya, Codogno, & Rodriguez‐Muela, 2018; Zhao, Fortier, & Baehrecke, 2018), and aging slows autophagy activity (Cuervo, 2008). Together, these results suggest that preventing production of fragmented mitochondria or clearing dysfunctional mitochondria during aging may promote GSC maintenance.

## 3 DISCUSSION

Here, we have described a shift of mitochondrial dynamics toward fission that occurs in GSCs during aging. This shift occurs in parallel with changes in factors that control stem cell self‐renewal and contributes to the loss (differentiation) of GSCs. In the GSCs of young germaria, the balance of mitochondrial dynamics favors fusion, resulting in mitochondria that are relatively elongated, healthy, and functional. Under these conditions, GSCs are well maintained. Blocked mitochondrial fusion in GSCs results in highly fragmented unhealthy mitochondria with decreased membrane potential, ROS generation, fatty acid metabolism defects, and attenuated BMP stemness signaling. Such GSCs divide slowly and are quickly lost from the niche. Interestingly, promoting fatty acid reentry into mitochondria partially restores the GSC loss phenotype when *marf* is depleted, indicating a role for lipid metabolism in GSC maintenance. Furthermore, preventing mitochondrial fission in GSCs causes cells to accumulate elongated healthy mitochondria, which may result in slightly increased ROS generation and stronger E‐cadherin‐mediated GSC attachment to the niche. These GSCs exhibited higher competiveness for niche occupancy. In aged GSCs, increased expression of Drp1 leads to increased mitochondrial fission accompanied by low mitochondrial membrane potential, decreased ROS levels, and reduced BMP signaling. Moreover, removed fragmented mitochondria in aged GSCs enhance their maintenance, preventing their loss during aging. Our results indicate that aging alters mitochondrial dynamics, shifting the balance from fusion to fission, and contributing to stem cell loss. These results may have broad significance for stem cell aging, particularly in light of the well‐known effects of aging on tissue degeneration and the close coupling of mitochondria with cellular metabolism.

The interplay between aging and mitochondrial dynamics has been previously studied in various laboratory models. For example, in *Drosophila*, promoting Drp1 expression or mitophagy in midlife flies extends lifespan, with mitochondria in the muscles of those flies appearing more fragmented compared to controls (Aparicio, Rana, & Walker, 2019; Rana et al., 2017). In a *C*.* elegans* study (Morsci, Hall, Driscoll, & Sheng, 2016), it was revealed that mitochondria size and density in neurons are increased in midlife but decreased to early‐life levels in aged worms, while promotion of mitochondrial fusion by AMPK or dietary restriction extends lifespan (Weir et al., 2017). Similarly, in fungal models, reducing mitochondrial fission also extends lifespan (Scheckhuber et al., 2007). Thus, manipulation of mitochondrial dynamics appears to be globally related to aging in tissues across many organisms. However, it is not clear whether all tissues show similar shifts in preference of mitochondrial dynamics during aging, and it is also unknown whether age‐related phasic patterns of mitochondrial dynamics happen in all cell types and in higher organisms. In mammals, this issue appears to be somewhat complicated. In studies on skeletal muscle, both fragmented mitochondria and large, less circular mitochondria have been reported in aged tissues (Beregi, Regius, Huttl, & Gobl, 1988; Iqbal, Ostojic, Singh, Joseph, & Hood, 2013; Leduc‐Gaudet et al., 2015). Furthermore, reducing mitochondrial fission partially reduces neurodegeneration in mouse neuronal disease models (Manczak, Kandimalla, Fry, Sesaki, & Reddy, 2016), while promoting mitochondrial fission reverses certain degenerative phenotypes in *Drosophila parkin* and *park* mutants (Deng, Dodson, Huang, & Guo, 2008; Yang et al., 2008). These species and tissue‐dependent results illustrate the importance of studies that experimentally delineate the mitochondrial profile during aging in various cellular contexts, which may aid the discovery of factors that influence long‐term mitochondrial maintenance.

### 3.1 Age‐dependent GSC loss is not due to defective fatty acid metabolism or increased ROS levels

An important function of mitochondria is to carry out long‐chain fatty acid oxidation for energy production. Forcing mitochondrial fragmentation in ovarian GSCs by disrupting *marf* results in reduced mitochondrial membrane potential and accumulation of oil droplets, which mostly contain stores of triglycerides composed of glycerol and three fatty acids. This droplet accumulation is not found in *drp1*‐depleted GSCs with elongated mitochondria. Similar results were recently reported in *Drosophila* male GSCs (Sênos Demarco et al., 2019). Interestingly, stimulating the reentry of accumulated lipids into mitochondria for oxidation by feeding *nos*>*marf^RNAi^* male and female flies with L‐carnitine decreases lipid accumulation and restores the GSC loss phenotype, indicating a contribution of fatty acid oxidation on GSC maintenance. In the study on *marf*‐defective male GSCs (Sênos Demarco et al., 2019), autophagy is found to be inhibited via TOR signaling, which is activated by excessive buildup of cytoplasmic fatty acids due to dysfunctional mitochondria. Removal of accumulated fatty acids or suppression of TOR activity promotes autophagy and prevents loss of *marf*‐defective male GSCs.

Although aged GSCs display more fragmented mitochondria and decreased mitochondrial membrane potential, we did not observe accumulation of oil droplets in these cells. This difference with *marf* mutant GSCs could be explained if only a subset of mitochondria are fragmented or if aged flies produce or consume low amounts of fatty acids. Nevertheless, feeding aged flies with rapamycin, which suppresses TOR activity to increase autophagy (Neufeld, 2010), significantly delayed GSC loss. This result suggests that inducing autophagy may help to clear fragmented mitochondria from aged GSCs, meaning the underlying mechanisms of GSC loss in aged *marf*‐defective and aged GSCs may be at least partially different.

According to the free radical theory of aging, ROS induces oxidative damage and leads to cellular dysfunction and aging (Barja, 2013). In support of this idea, damaged mitochondria and ROS levels are known to be increased in aged fly and mammalian brains (Chakrabarti et al., 2011; Scialo et al., 2016). Surprisingly, in aged GSCs from female *Drosophila*, there is an approximately 14% reduction in ROS levels compared to young GSCs. Nevertheless, constitutive overexpression of superoxide dismutase (SOD; helps remove ROS) delays GSC loss during aging (Pan et al., 2007). These observations might be reconciled if SOD expression keeps ROS levels low throughout the entire lifespan of the GSC. Further analysis of ROS levels in aged GSCs from male flies or GSCs in other species will help to determine whether and how the free radical theory of aging may be applicable in a cell type‐specific manner.

### 3.2 Mitochondrial dynamics control germ cell differentiation

Several lines of evidence have shown that mitochondrial dynamics control cell fate decisions (Bahat & Gross, 2019; Chen & Chan, 2017), including in stem cells. For example, Drp1‐induced mitochondrial fission is required for Notch‐mediated follicle cell differentiation during *Drosophila* oogenesis (Mitra, Rikhy, Lilly, & Lippincott‐Schwartz, 2012). Mouse embryonic neural stem cells carry elongated mitochondria and rely on glycolysis for energy production, and enhanced mitochondrial fragmentation promotes the commitment of neural stem cells to differentiation and maturation (Khacho et al., 2016). However, the role of mitochondrial dynamics in germ cell differentiation is less clear.

In *Drosophila* female GSCs, mitochondria are elongated and form clusters near the fusome (see Figure 1); similar mitochondrial morphology is also observed in the immediate daughter cells, CBs. Mitochondria are highly fragmented in 4‐ and 8‐cell cysts (see Figure 4), suggesting that fission is preferred during differentiation; meanwhile, mitochondria are elongated again in 16‐cell cysts (see Figure 4), which are undergoing meiosis (Lin & Spradling, 1993). These observations are in agreement with a previous study (Cox & Spradling, 2003). In this study, we show that forcing mitochondria fragmentation in a *marf* mutant promotes GSC differentiation, and aging‐associated mitochondrial fragmentation contributes to age‐dependent GSC loss, at least in part via the upregulation of Drp1. Although Marf is also required for *Drosophila* male GSC maintenance (Sênos Demarco et al., 2019), the balance of mitochondrial dynamics in young and aged male GSCs is not clear. Interestingly, mitochondria in *drp1* mutant 4‐ or 8‐cell cysts are still highly fragmented (dashed outline in Figure S8A), suggesting that additional factors regulate mitochondrial fission in germ cell cysts. Furthermore, we often observed *drp1*‐mutant GSCs overloaded with mitochondria with proximal CBs only carrying a few mitochondria or vice versa (Figure S8B,C). This observation suggests that mitochondrial fission is necessary for proper partitioning of mitochondrial mass into the two daughter cells during cell division (Mishra & Chan, 2014). Occasionally, we found CBs or 2‐cell cysts with very few mitochondria positioned in the posterior germarium (Figure S8D), implying that these cells may be defective in differentiation; perhaps cellular metabolism cannot meet the energetic requirements for differentiation in such cells. Mitochondria elongation mediated by Mfn1 and Mfn2 (Pernas & Scorrano, 2016) was shown to be critical for efficient ATP production via oxidative phosphorylation during mouse spermatogonial differentiation and a metabolic shift that occurs during meiosis (Varuzhanyan et al., 2019). Similarly, mitochondrial maturation with maximal ATP and ROS generation also promotes GSC differentiation and oocyte formation in *C*.* elegans* (Charmpilas & Tavernarakis, 2020). These studies combined with our results suggest a conserved role for mitochondrial dynamics in germ cell fate decisions among species. Importantly, physiological aging drives a shift of mitochondrial dynamics toward fission in *Drosophila* female GSCs, an observation that might be generalizable to stem cells in different tissues.

## 4 MATERIALS AND METHODS

### 4.1 *Drosophila* strains and culture

*Drosophila* stocks were maintained at 22–25°C on standard medium, unless otherwise indicated. For aging experiments, flies were fed with normal diet plus a paste of wet yeast and food was changed daily. *yw* was used as a wild‐type control. The following fly strains were used in this study: Hypomorphic *marf^E^* and null *bam^△86^*, *bam^1^*, *marf^B^*, *drp1^1^*, and *drp1^2^* alleles have been described previously (*marf* and *drp1* mutant fly lines are kind gifts from Dr. Hugo Bellen, Department of Molecular and Human Genetics, Baylor College of Medicine) (Gonczy, Matunis, & DiNardo, 1997; McKearin & Spradling, 1990; Sandoval et al., 2014). *UASp*‐*mito*‐*gfp*, that is, human COX VIII mitochondrial targeting signal fused to the N‐terminus of EGFP, was used to monitor mitochondria (a kind gift from Dr. Allan C. Spradling, Department of Embryology, Carnegie Institution of Science) (Cox & Spradling, 2003). *pCasper*‐*mfn*‐*egfp*, a transgene construct with GFP‐tagged Mfn under the control of the endogenous Mfn promoter, was used to monitor the endogenous Mfn level (Zhang et al., 2017); an appropriate anti‐Marf antibody for immunostaining is lacking. *UAS*‐*RNAi* lines against *marf* (BL55189) and *drp1* (BL51483) were obtained from the Bloomington *Drosophila* Stock Center (BL). The efficiencies were described previously (Deng et al., 2016; Smith et al., 2019) and were tested again in this study (Figure S4). The *nos*‐*Vp16*‐*GAL4* line was used to drive *RNAi* expression in the germline line (Doren, Williamson, & Lehmann, 1998; Tseng et al., 2014). Other genetic elements are described in FlyBase (http://flybase.bio.indiana.edu). Flies expressing *RNAi* or *mito*‐*gfp* by *nos*‐*GAL4* were cultured at 18°C to suppress GAL4 activity during developmental stages; the flies were switched to 29°C after eclosion to activate GAL4. For the egg laying assay, five 2‐day‐old females were cultured with five *w^1118^* males for 1 day at 25°C (the experiment was performed in triplicate), then transferred into plastic bottles with small holes at the bottom and capped by a molasses plate supplied with a wet yeast paste. The bottles were placed upside‐down, and the molasses plate was changed daily. Numbers of eggs on the molasses petri dish were counted.

### 4.2 Genetic mosaic analysis

Genetic mosaics were generated by FLP/FRT‐mediated mitotic recombination (Xu & Rubin, 1993). Three‐ to 5‐day‐old flies of the genotypes *FRT19A*/*ubi*‐*gfpFLP122FRT19A*, marf*FRT19A/*ubi*‐*gfpFLP122FRT19A*, *hs*‐*flp*/+;* FRT40A*/*ubi*‐*gfpFRT40A*, *hs*‐*flp*/+; drp1*FRT40A/*ubi*‐*gfpFRT40A* and marf*FRT19A/*ubi*‐*gfpFLP122FRT19A*; drp*FRT40A/*arm*‐*lacZFRT40A* (*represents *marf* or *drp1* mutant alleles) were generated from standard crosses and subjected to heat shock for 1 hr at 37°C, twice a day for 3 days. After heat shock, flies were cultured at 25°C and food was changed daily until dissection. Homozygous mutant cells were identified by the absence of GFP. For flip‐out clone analysis, 5 and 8‐week‐old flies of genotypes *hs*‐*flp*/+;* nos *≫ *mcherryV40ply*‐*STOP *> *GAL4UAS*‐*gfp*/*UAS*‐*drp1^RNAi^*, and *hs*‐*flp*/+;* nos *≫ *mcherryV40ply*‐*STOP *> *GAL4UAS*‐*GFP*/+;* UAS*‐*mcd8*‐*gfp*/+ were generated from standard crosses and subjected to heat shock one or 2 weeks prior to dissection for 30 min at 37°C for 3 times a day for 3 days. After heat shock, flies were cultured at 25°C until dissection. Food with wet yeast paste was changed daily. RNAi‐expressing cells were recognized by the presence of GFP in flip‐out clones.

### 4.3 Immunostaining and fluorescence microscopy

Ovaries were dissected, fixed, and immunostained as described (Yang et al., 2013). In brief, ovaries were dissected in pre‐warmed Grace's insect medium (GIM, Lonza) and fixed with 5.3% paraformaldehyde/GIM for 13 min with gentle agitation at room temperature. Ovaries were washed in PBST (0.1% Triton X‐100 in 1X PBS) for 20 min three times and teased apart in PBST before incubating with blocking solution (5% bovine serum albumin (BSA) and 0.05% normal goat serum in PBST) for 3 hr at room temperature or 4°C overnight. Ovaries were incubated with primary antibodies (diluted in blocking solution) for 3 hr at room temperature or 4°C overnight, followed by three or four 30‐min washes with PBST. Next, ovaries were incubated with secondary antibodies (diluted in blocking solution) for 3 hr at room temperature or 4°C overnight, followed by three or four 30‐min washes with PBST. Apoptag^®^ Fluorescein *In Situ* Apoptosis Detection Kit (cat#S7110, Merck) was used to detect apoptotic cells following the instruction manual with slight modifications (Tseng et al., 2014). In brief, ovaries were fixed, teased apart, and incubated with 300 µl of equilibration buffer for 5 min on rotator twice at room temperature. Ovaries were then incubated with a reaction mix consisting of 76 µl reaction buffer and 32 µl TdT enzyme for 1 hr at 37°C in a dark chamber. The reaction was stopped by adding 500 µl STOP/WASH solution, and samples were subsequently rinsed with PBST for 20 min before further staining. The following primary antibodies were used: mouse anti‐Hu‐li tai shao (*Drosophila* adducing‐related protein) (1:25; 1B1, Developmental Studies Hybridoma Bank, DSHB), mouse anti‐Lamin C (1:25; LC28.26, DSHB), rabbit anti‐GFP (1:1000; cat#TP401, Torrey Pines), rabbit anti‐Drp1 (1:100, a gift from Dr. Leo J. Pallanck, Department of Genome Sciences, University of Washington), rat anti‐DE‐cadherin (1:3, DCAD2, DSHB), rabbit anti‐Smad3 (phospho S423+S425) (1:250, ab52903, Abcam), mouse anti‐ATP5ase (1:1000, 15H4C4, Abcam), and mouse anti‐mono‐and poly‐ubiquitinylated conjugates (1:100, FK2, Enzo). Alexa Fluor 488‐ or 568‐ or 633‐conjugated goat anti‐mouse, anti‐rabbit, anti‐rat, and anti‐chicken secondary antibodies (Molecular Probes or Abcam, 1:500) were used. Samples were stained with 0.5 μg/ml DAPI (Sigma), followed by mounting in mounting solution [80% glycerol containing 20.0 µg/ml N‐propyl gallate (Sigma)]. Images of fixed ovaries were obtained using a Zeiss LSM 700 Laser Scanning confocal microscopes.

### 4.4 BODIPY 493/503 staining

Ovaries were dissected, and immunostaining was performed as described above. After immunostaining, ovaries were incubated with 50 μM BODIPI 493/503 (D3922, Thermo Fisher) in 0.1% PBST in the dark at RT for 20 min. Samples were washed 3 times with 0.1% PBST, stained with 0.5 μg/ml DAPI for 5 min, and mounted in mounting solution, as described above.

### 4.5 L‐Carnitine and rapamycin treatment

L‐carnitine (C0283‐5G, Thermo Fisher, final concentration of 25 mg/ml) or rapamycin (R0395, Sigma‐Aldrich, final concentration of 200 μM) were added to wet yeast (yeast to water ratio was 1.8 g:1 ml). One‐week‐old flies were fed wet yeast with or without L‐carnitine (or rapamycin) in vials containing molasses food for 1 week. Food was changed every day until dissection.

### 4.6 Image analysis

GSCs were identified by the position of the fusome (labeled by 1B1 staining), which is adjacent to cap cells (cap cell nuclear envelopes were labeled by LamC staining) (Hsu & Drummond‐Barbosa, 2011). Germaria analyzed for GSC division and expression of pMad and E‐cadherin contained at least one GFP‐negative and one GFP‐positive GSC. To measure GSC relative division rates, the number of GFP‐positive progeny (cystoblasts and cysts) was divided by the number of GFP‐positive GSCs, and this value was divided by the number of GFP‐negative progeny divided by the number of GFP‐negative GSCs in a given germarium. Each CB undergoes four more rounds of division to form 2‐, 4‐, 8‐, and 16‐cell cysts, and the cells in each cyst remain interconnected by a branched fusome. Therefore, the number of fusomes represents the number of GFP‐negative progeny derived from a GFP‐negative GSC and likewise for the fusomes carried by GFP‐positive progeny. To measure pMad expression, Image J was used to quantify the average fluorescence intensity (arbitrary units) in confocal z‐sections at the largest GSC nuclear diameter. For E‐cadherin measurement, the z‐section with strongest E‐cadherin expression in the junction between cap cells and GSC was analyzed. For oil droplet analysis, number and size of BODIPY signals in each GSC cytoplasm were analyzed by ImageJ. For mitochondria analysis, images of germaria labeled for ATP5ase were deconvoluted using MetaMorph (Molecular Devices) and analyzed by Imaris.

### 4.7 Live imaging and image processing

Ovaries of 1‐ and 8‐week‐old *nos*>*mito*‐*gfp* flies were dissected in pre‐warmed GIM and stained with Hoechst (5 μg/ml) for 10 min at room temperature. Anterior ovarioles were dissected from Hoechst‐stained ovaries, amounted with CellTak (Corning) on 5‐mm‐diameter pre‐cleaned cover slips (Warner Instruments, 64‐0700), immersed in a PBS‐filled chamber, and imaged with lattice light‐sheet microscopy (Chen et al., 2014). Images were scanned with 100 z‐sections every ~2 s for 300 frames with a detection objective (Nikon, CFI Apo LWD 25XW, 1.1 NA, 2 mm WD) at a speed of 10 ms exposure per plane. Raw images taken from the light‐sheet microscope were deconvoluted by Amira (version 6.4, Thermo Fisher) with the PSF kernel acquired under the same optical condition. The deconvoluted images taken at the first 10 time points were stacked and corrected for bleaching to decrease background using ImageJ (Fiji, NIH). For each stack, the anterior of the germarium was cropped and subjected to Amira (Thermo Fisher) segmentation in order to define mitochondria by “hysteresis thresholding.” Each stack with defined mitochondria was analyzed with Imaris (Bitplane) to track mitochondrial dynamics within time points 1‐4, 4‐7, and 7‐10. Mitochondrial fusion/fission was tracked using the connected components model (Dillencourt, Samet, & Tamminen, 1992). By this model, spots in adjacent time points are considered connected if the spot spheres occupy some of the same space (the spheres would overlap if the two time points were merged into one image). This method essentially compares the amount of overlap between identified objects in the previous frame with those in the current frame. All objects with overlap are assigned the same track ID. For Mitotracker staining, ovaries were dissected in pre‐warmed GIM; 5 pairs of ovaries were dissected within 10 min. Ovaries were then incubated with Mitotracker Red (1:3,000, Molecular Probes) in the GIM at 25°C, gently rotating for 30 min, avoiding light. The solution was later replaced with pre‐warmed GIM to clear the background. The ovaries were mounted in pre‐warmed GIM. The anterior‐most germ cells in the germarium were considered to be GSCs in live ovarioles, as it was not feasible to label GSC fusome and niche cells. Images of live ovaries were obtained using a Zeiss LSM 700 Laser Scanning confocal microscopes.

### 4.8 Transmission electron microscopy (TEM) of adult germaria

Ovaries of were dissected in 2.5% glutaraldehyde/2% paraformaldehyde/1% tannic acid/0.1 M sodium cacodylate buffer and fixed in buffer at 4°C for overnight. Ovaries were washed for 10 min three times in 0.2 M sucrose/0.1% CaCl_2_/0.1 M sodium cacodylate buffer at 4°C and incubated for 2 hr in 1% OsO_4_, 0.1 M cacodylate buffer at room temperature. After rinsing with cold H2O three times at 4°C (Each time for 5 min), ovaries were immersed in 1% uranyl acetate for 1 hr at 4°C. Ovaries were dehydrated through a gradient of ethanol concentrations [30%, 50%, 75%, 90% for 1 time and 100% for 3 times (each time for 5 min)] at 4°C and infiltrated with Spurr's Resin (the Low Viscosity Embedding Media Spurr's Kit, Electron Microscopy Sciences) with ethanol ratios of 1:3, 1:1, and 3:1 and then with pure Spurr's Resin for times (each time for 1 hr). Finally, ovaries were polymerized at 60°C for 48 hr. Ultrathin sections were sectioned with a diamond knife (DiATOME) on a microtome and stained with 4% uranyl acetate for 20 min. After rinsing with H_2_O six times, ovaries were immersed in Reynolds lead citrate for 10 min. Then, slices were rinsed with H_2_O and mounted on copper slot grids and observed under TEM. After mounting, germarial sections were examined with a Tecnai G2 spirit TWIN transmission electron microscope (FEI Company) equipped with a Multiscan Gatan camera (Gatan) at an accelerating voltage of 120 kV. Finally, images were subjected to analysis of mitochondria using ImageJ and Amira software or movie production by Tomographic 3D Image Reconstruction.

### 4.9 Flow cytometry‐based mitochondrial membrane potential and ROS measurement

GSC dissociation for flow cytometry analysis was performed as previously described (Kao et al., 2015). In brief, flies with the genotypes, *UAS*‐*marf^RNAi^*/+; *vasa*‐*gfpbam^△86^*/*nos*‐*GAL4bam^1^*, *UAS*‐*drp1^RNAi^*/+;* vasa*‐*gfpbam^△86^*/*nos*‐*GAL4bam^1^*, *vasa*‐*gfpbam^△86^*/*nos*‐*GAL4bam^1^*,*bam^△86^*/*nos*‐*GAL4bam^1^* were grown at 18°C and shifted to 29°C after eclosion. Seven to twelve pairs of ovaries from each genotype were dissected in pre‐warmed GIM plus 10% FBS (GIM‐FBS) and were subsequently incubated with 0.45% Trypsin (Solution 10X, cat# 9002077, Sigma‐Aldrich) and 2.5 mg/ml collagenase (cat#17018‐029, Gibco) on a rotator at 25°C for 25 min with vigorous shaking; samples were vortexed every 5 min. Digested ovaries were filtered through a 40‐µm nylon mesh and then centrifuged at 1,000 *g* for 7 min to harvest the cell pellet. The pellets were resuspended in 500 µl GIM‐FBS containing 10 nM membrane potential probe TMRE (cat#T669, Thermo Fisher)/or 30 µM ROS probe DHE (cat#D11347, Invitrogen) and 0.5 µg/ml of DAPI with vigorous shaking at RT for 10 min in a dark chamber. For a positive control of membrane potential measurement, cells dissociated from *bam^△86^vasa*‐*gfp*/*nos*‐*GAL4bam^1^* ovaries were co‐treated with 10 nM TMRE and 10 µM FCCP (to depolarize the mitochondrial membrane, C2920, Sigma‐Aldrich) for 10 min under the same conditions as described above. For a positive control of ROS measurement, cells dissociated from *bam^△86^vasa*‐*gfp*/*nos*‐*GAL4bam^1^* ovaries were treated with 30 µM DHE and 100 µM paraquat (to induce cellular ROS, cat#3752782, Sigma‐Aldrich) for 10 min under the same conditions as described above. The stained cells were detected using an Attune NxT acoustic focusing cytometer (Thermo Fisher Scientific) at 480/530, 405/440, 480/590, and 561/585 (Excitation/Emission) to measure GSCs carrying vasa‐GFP, DAPI‐labeled dead cell, DHE, and TMRE, respectively. TMRE/or DHE intensity was measured from GFP‐positive and DAPI‐negative GSCs, and at least 10,000 GSCs’ intensity was measured and averaged for one replicate; three replicate were done for each measurement.

## CONFLICT OF INTEREST

The authors declare that there is no conflict of interest regarding the publication of this article.

## AUTHOR CONTRIBUTIONS

O.A., C.‐H.L., A.C, and H.‐J. H. designed and interpreted the experiments and wrote the paper. C.‐H. L., S.‐H. K., and O.A. contributed to TEM image analysis; B.‐C. C., C.‐H. L., S. ‐C. H. W.‐C. T., and S.‐H. K. contributed to live image recording and analysis; C.‐K. Y. and G‐C. C. provided reagents; H‐L.C performed egg laying assay; D.L.C and Y. Y. H performed LD analysis; B.‐S. H. performed Mitotracker staining; A.C. and E. R. counted GSC number; K.‐Y. L. and Y.‐T. W. provided valuable comments and discussion. O.A. performed the remaining experiments.

## Supporting information

Figure S1

Figure S2

Figure S3

Figure S4

Figure S5

Figure S6

Figure S7

Figure S8

Video S1

Video S2

Video S3

Supplementary Material

Table S1

## Data Availability

The data that support the findings of this study are available upon request.
