# Supplementary figures and images for: Aging shifts mitochondrial dynamics toward fission to promote germline stem cell loss
Source: Aging Cell. 2020 Jul 14;19(8):e13191. doi: 10.1111/acel.13191 (PMC7431834; doi:10.1111/acel.13191)

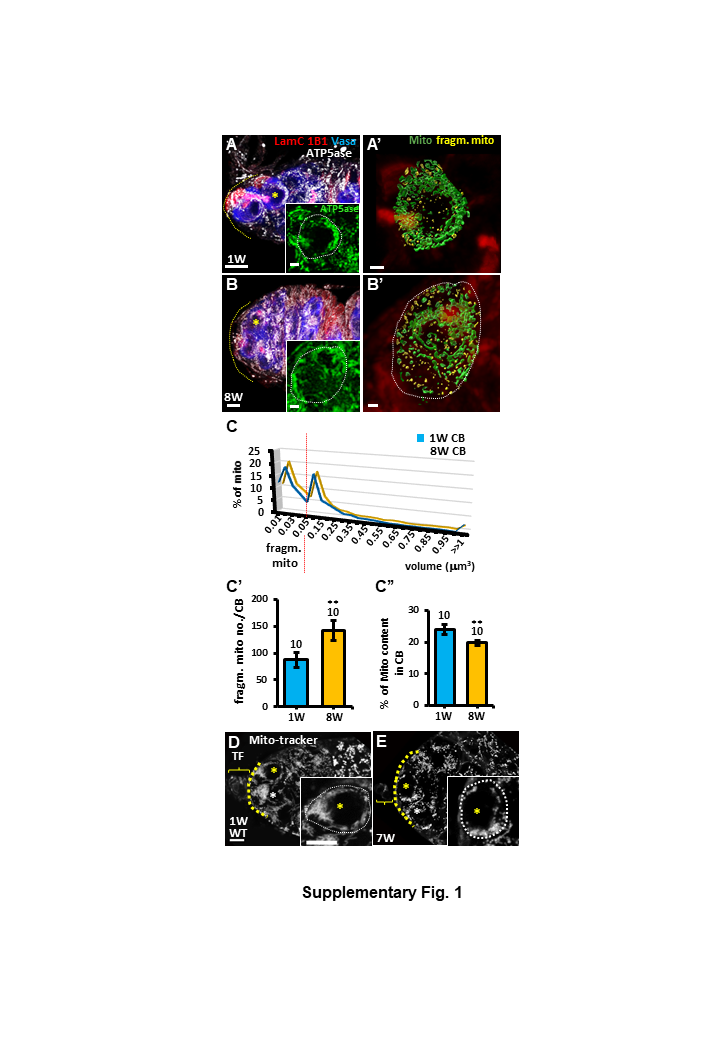

Supplement: Supplementary file 1 — Figure S1 [file ACEL-19-e13191-s001.TIF]

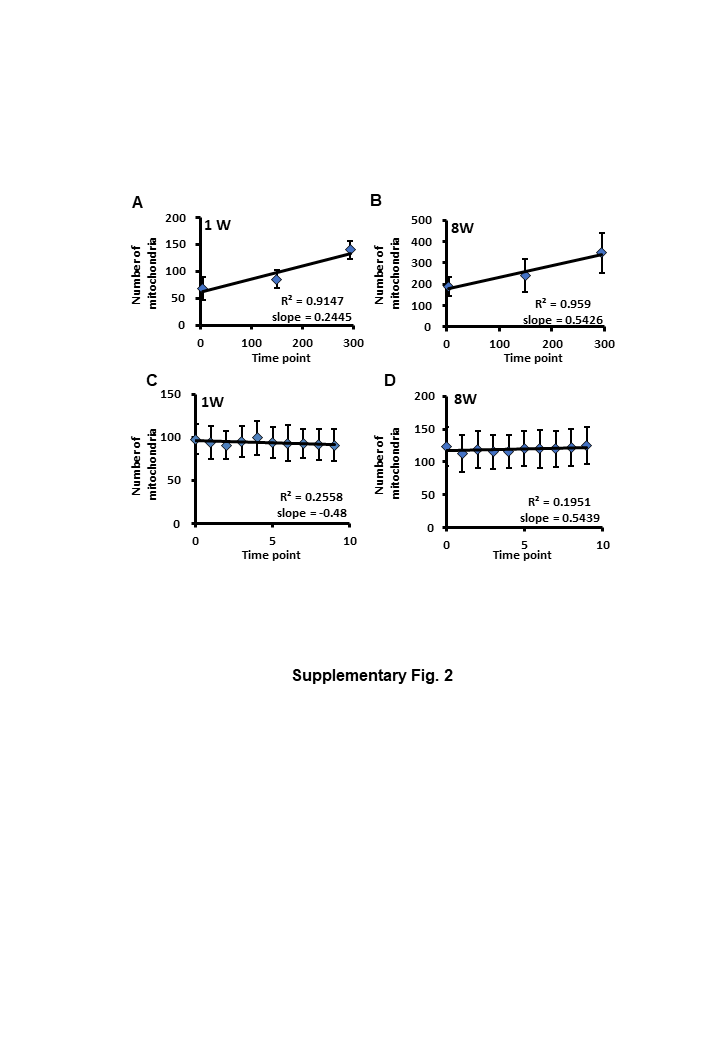

Supplement: Supplementary file 2 — Figure S2 [file ACEL-19-e13191-s002.TIF]

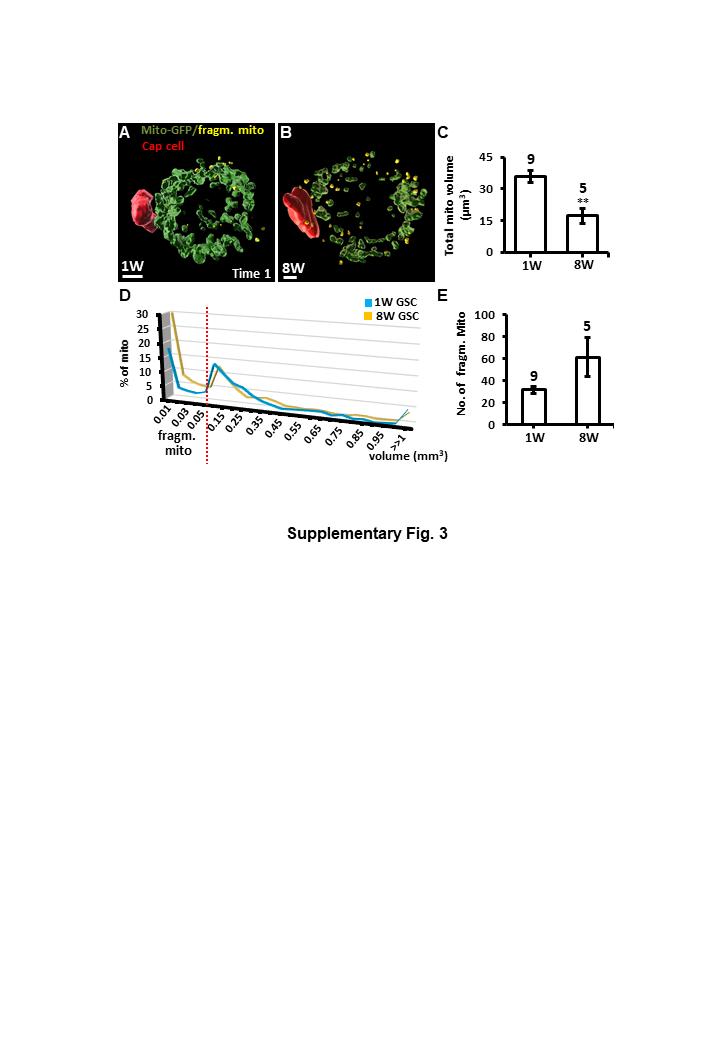

Supplement: Supplementary file 3 — Figure S3 [file ACEL-19-e13191-s003.TIF]

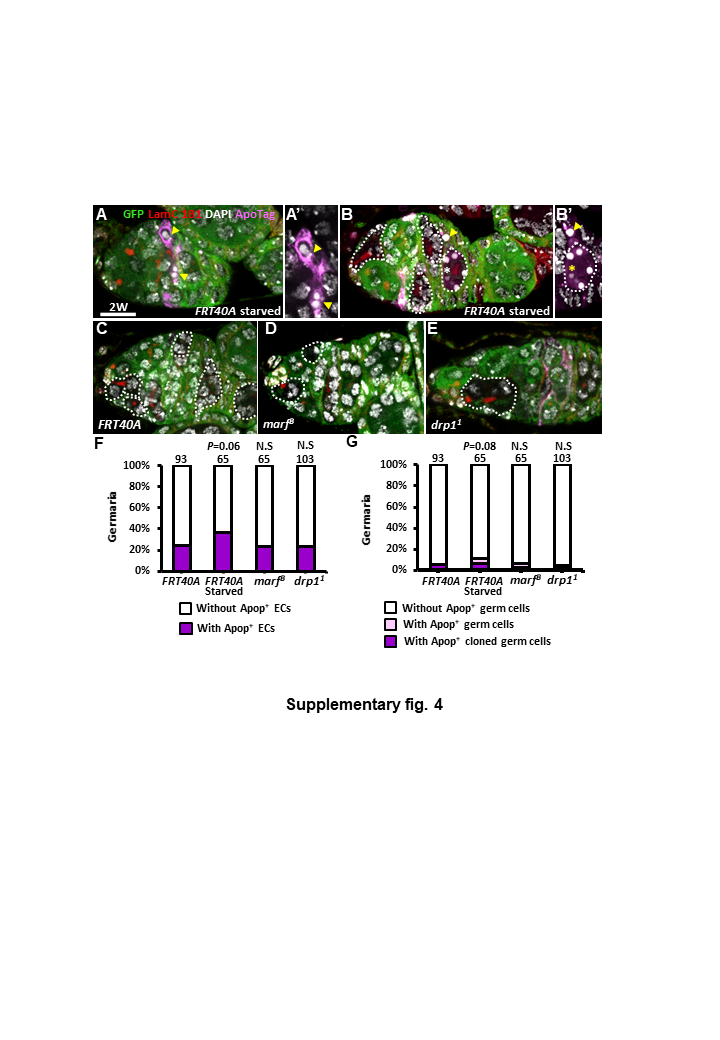

Supplement: Supplementary file 4 — Figure S4 [file ACEL-19-e13191-s004.TIF]

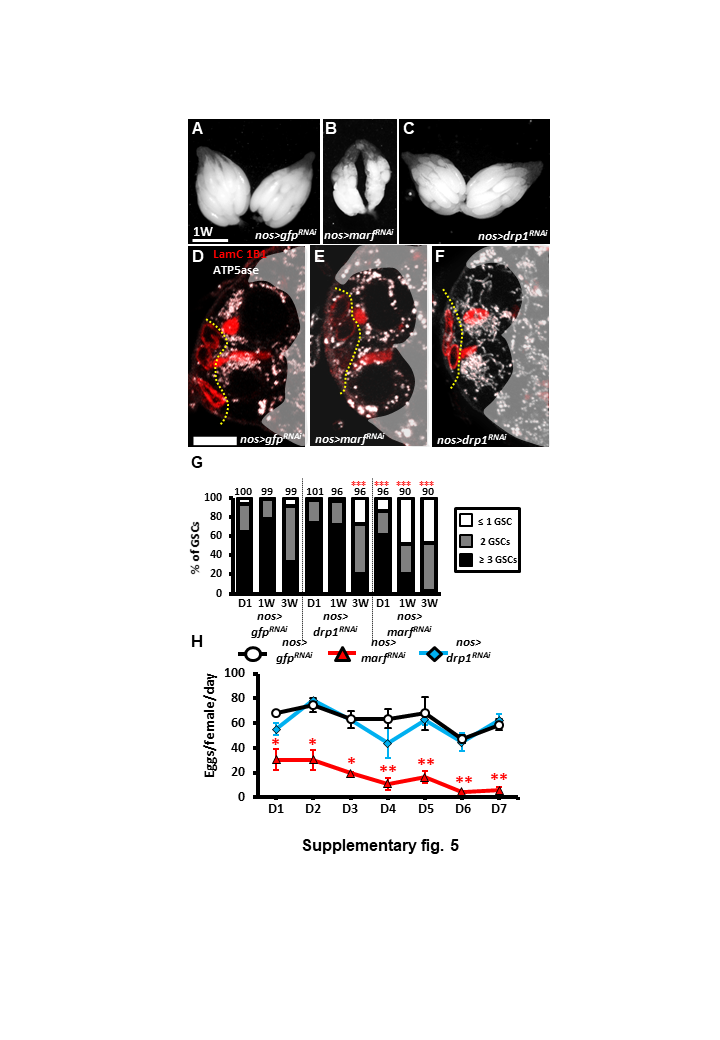

Supplement: Supplementary file 5 — Figure S5 [file ACEL-19-e13191-s005.TIF]

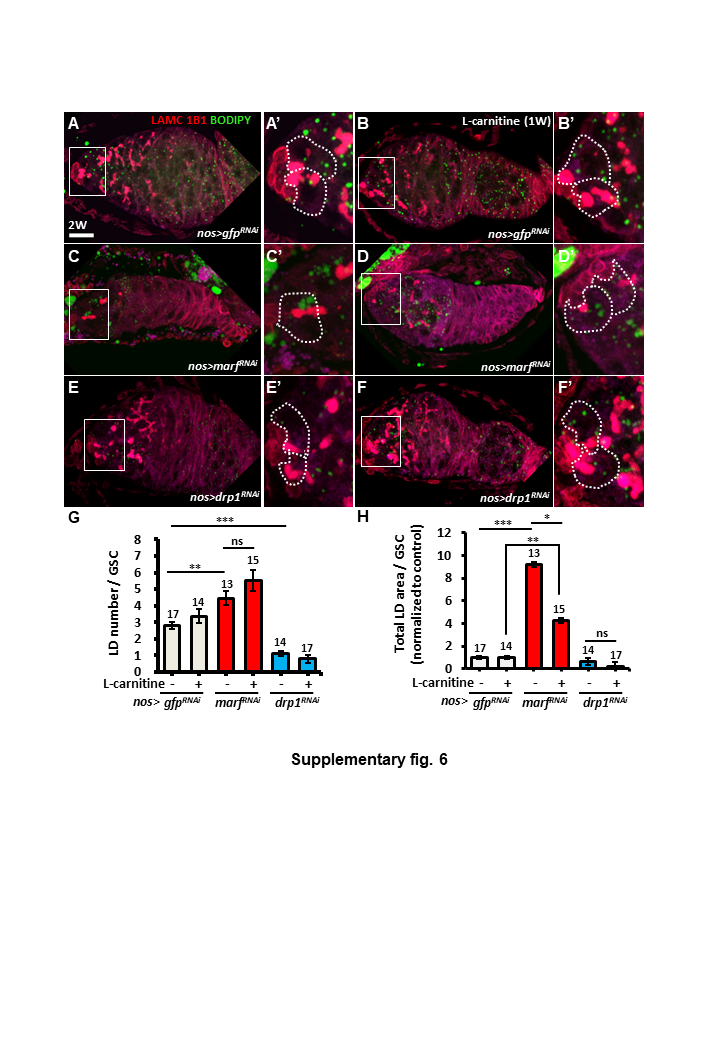

Supplement: Supplementary file 6 — Figure S6 [file ACEL-19-e13191-s006.TIF]

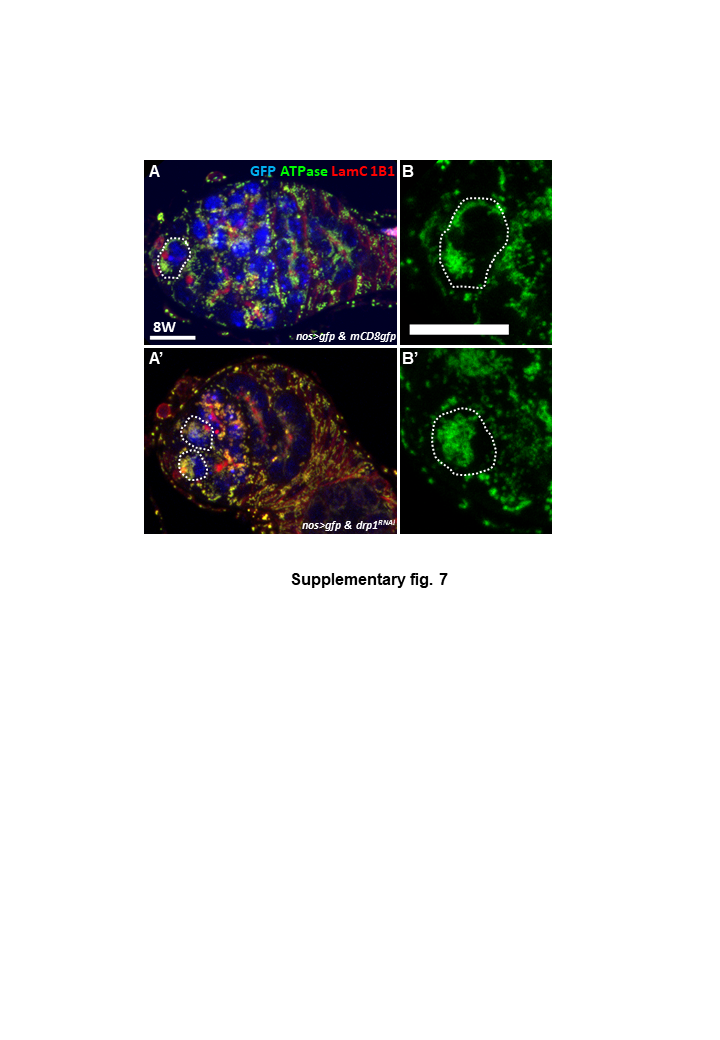

Supplement: Supplementary file 7 — Figure S7 [file ACEL-19-e13191-s007.TIF]

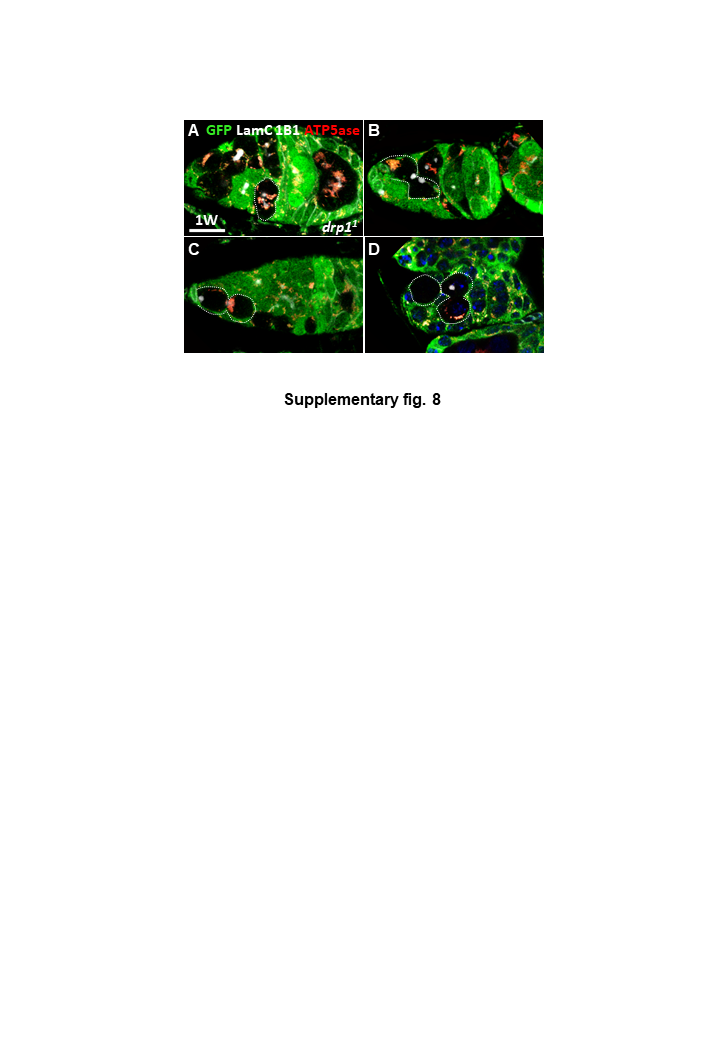

Supplement: Supplementary file 8 — Figure S8 [file ACEL-19-e13191-s008.TIF]
