# Supplementary material for: Aging shifts mitochondrial dynamics toward fission to promote germline stem cell loss
Source: Aging Cell. 2020 Jul 14;19(8):e13191. doi: 10.1111/acel.13191 (PMC7431834; doi:10.1111/acel.13191)
Supplement: Supplementary file 12 — Supplementary Material [file ACEL-19-e13191-s012.docx]

**Supporting information**

**Supplementary Fig. 1-8**

**Supplementary Table 1. Mitochondrial dynamics controls GSC maintenance.**

**Supplementary movie 1. Mitochondrial dynamics in young GSCs.** Mitochondria were tracked in live one-week old *nos>mito-gfp* GSCs for the first 10 time points (interval is ~2 sec). Germaria were stained with permeable DNA dye (Hoechst) for cap cell recognition based on cap cell nuclear size and morphology, as well as cap cell location. Only Hoechst signals in cap cells were kept, and Hoechst signals in other cell types were omitted. Only Mitochondria and cap cells are displayed as a surface-rendered 3D trace. Mitochondria and cap cells are displayed as a surface-rendered 3D trace. Purple, mitochondria; yellow, fragmented mitochondria, red: cap cells. Scale bar, 2 μm.

**Supplementary movie 2. Mitochondrial dynamics in aged GSCs.** Mitochondria were tracked in live 8-week old *nos>mito-gfp* GSCs for the first 10 time points (interval is ~2 sec). Germaria were stained with permeable DNA dye (Hoechst) for cap cell recognition based on cap cell nuclear size and morphology, as well as cap cell location. Only Hoechst signals in cap cells were kept, and Hoechst signals in other cell types were omitted. Only Mitochondria and cap cells are displayed as a surface-rendered 3D trace. Purple, mitochondria; yellow, fragmented mitochondria, red: cap cells. Scale bar, 2 μm.

**Supplementary movie 3. Mitochondrial dynamic in the one-week-old GSC within the time point 1-4.**  Mitochondria were tracked in live one-week old *nos>mito-gfp* GSCs for the first 10 time points (interval is ~2 sec). Germaria were stained with permeable DNA dye (Hoechst) for cap cell recognition based on cap cell nuclear size and morphology, as well as cap cell location. Only Hoechst signals in cap cells were kept, and displayed as a surface-rendered 3D trace. Gray, mitochondria; red, cap cells. Scale bar, 1 μm.

**Supplementary figure legends**

**Supplementary fig. 1 Aged GSCs and cystoblasts exhibit increased fragmented mitochondria. (A and B)** One- (A) and 8-week-old germaria (B) with LamC (red, TF and cap cell nuclear envelopes), 1B1 (red, fusomes), Vasa (blue, germ cells), and ATP5ase (gray, mitochondria). Inserts are higher magnification of cytoblast (CBs) marked by yellow asterisks in E and F, with ATP5ase shown in green. (A’ and B’) are same CBs shown in the inserts, but with less layers and the surface model of mitochondria from Imaris shown. Mitochondria (mito) forming networks are shown in green; fragmented mitochondria (fragm. mito) are shown in yellow. Asterisks indicate CB(s) in the germarium; dashed circles outline CBs. Yellow dashed lines outline the anterior edge of the germarium. Scale bars in A and B are 5 μm, in the insert of A and B are 2 μm, and in A’and B’ are 1μm. **(C)** Percentage (%) of mitochondria with indicated volume in 1- and 8-week-old CBs. **(C’)** Number of fragmented mitochondria in 1- and 8-week-old CBs. **(C’’**) Percentage of mitochondrial content per GSC in 1- and 8-week-day-old CBs. Number of analyzed CBs are shown above each bar. Error bars, SEM.*, *P* < 0.05; ***, *P* < 0.001. **(D and E)** One- (D) and 7-week (W)-old live germaria (E) with mitotracker (gray, mitochondria). Inserts are higher magnification of GSCs marked by yellow asterisks in D and E. Scale bar is 5 μm. Representative germaria are shown in 3D-reconstructed images; genotype of flies is *yw*.

**Supplementary fig. 2 Long laser exposure times increase mitochondria number.** Number of mitochondria in one- (A and C) and 8-week-old live GSCs (B and D) recorded for 300 time points (A and B) and 10 time points (C and D). The interval between each time point is 2 sec.

**Supplementary fig. 3 Aged GSCs display decreased mitochondrial content and increased numbers of fragmented mitochondria. (A and B)** Mito-GFP-marked mitochondria (magenta) in 1- (A) and 8-week (W)-old live GSCs (B) with cap cells (red, marked by Hoechst labeling) displayed as surface-rendered 3D models. The cap cell can be recognized by its small ovoid nucleus (red) and proximity to GSCs. Fragmented mitochondria are shown in yellow. Scale bar, 2 μm. **(C)** Total mitochondrial volume in 1- and 8-week-old live GSCs at the first time point of the live imaging. **(D)** Percentage (%) of accumulated mitochondria with the indicated mitochondrial volume in 1 and 8-week-old GSCs. **(E)** Number (no.) of fragmented mitochondria (volume smaller than 0.05 μm^3^) in 1- and 8-week-old live GSCs at the first time point of the live imaging. Numbers of analyzed GSCs are shown above each bar. **, *P* < 0.01. Error bars, mean ± SD.

**Supplementary fig. 4 Disruption of mitochondrial dynamics in germ cells does not cause germ cell death. (A-E)** *FRT40A* control (ctrl) under starvation (A and B), *FRT40A* control (C), *marf^B^* (D) and *drp1^1^* mosaic germaria (E) with GFP (green, wild-type cells), 1B1 (red, fusomes), LamC (red, cap cell nuclear envelopes), and ApoTaq (magenta, apoptotic cells) labeling 2 weeks after clonal induction (ACI). Scale bar, 10 μm. A’ and B’ show enlarged views of apoptotic cells with DAPI and ApoTaq channels from A and B. Dashed lines outline germ cell clones; arrows indicate dying escort cells; asterisk marks dying germ cells. **(F and G)** Percentage (%) of germaria carrying apoptotic escort cells (F) and germ cells (G) with indicated genotypes under starvation or normal diet. Number of analyzed germaria are shown above each bar. N.S, no significant difference.

**Supplementary fig. 5 Decreasing expression of Drp1 in the germline decreases GSC maintenance and egg production. (A-C)** One-week (W)-old *nos>gfp^RNAi^* (A), *nos>marf^RNAi^* (B), and *nos>drp1^RNAi^* ovaries (C). Scale bar, 0.5 mm. **(D-F)** One-week -old *nos>gfp^RNAi^* (D), *nos>marf^RNAi^* (E), and *nos>drp1^RNAi^* germaria (F) with LamC (red, cap cell envelopes), 1B1 (red, fusomes) and ATP5ase (gray, mitochondria) labeling. Scale bar, 5 μm. **(G)** Percentage (%) of germaria carrying indicated GSC number in *nos>gfp^RNAi^*, *nos>marf^RNAi^*, and *nos>drp1^RNAi^* ovaries in newly eclosed (D) flies, or at 1 week and 3 weeks after eclosion. Numbers of analyzed germaria are shown above each bar. **(H)** Egg production assay with 2-day (D)-old *nos>gfp^RNAi^*, *nos>marf^RNAi^*, and *nos>drp1^RNAi^* females for 7 days. *, *P* < 0.05; **, *P* < 0.01; ***, *P* < 0.001. Error bars, mean ± SEM.

**Supplementary fig. 6 One-week treatment of L-carnitine reduces lipid droplet accumulation in GSCs induced by *marf*-knockdown. (A-F**) Two-week (W)-old *nos>gfp^RNAi^* (A and D), *nos>marf^RNAi^* (B and E), and *nos>drp1^RNAi^* germaria (C and F) without L-carnitine treatment (A-C) and treated with L-carnitine beginning at one week of age (B-F). Germaria with LamC (red, terminal filament and cap cell nuclear envelopes), 1B1 (red, fusomes and follicle cell membrane), and BODIPY 493/503 (green, lipid droplets (LD)). (A’-F’) Enlarged image containing GSCs. Scale bar, 5µm. (**G**) Number of lipid droplets per GSC of 2-week-old flies with indicated genotype, with and without L-carnitine treatment. (**H**) Total lipid droplet area per GSC (normalized to *nos>gfp^RNAi^* without treatment) of 2-week-old flies with indicated genotypes, with and without L-carnitine treatment. Numbers of analyzed GSCs are shown above each bar. *, *P* < 0.05; **, *P* < 0.01; ***, *P* < 0.001. Error bars, mean ± SEM.

**Supplementary fig. 7 Knockdown Drp1 in aged GSCs forms mitochondrial clustering.** A flip-out system was used for *nos-gal4*-driven *drp1* knockdown in aged GSCs; knockdowns are identified by the presence of GFP expression (blue). In females carrying a *nos* promoter-driven FRT-flanked flip-in *GAL4/VP16* construct (*nos>STOP>GAL4*), GAL4 is not expressed unless the stop cassette was removed by Flipase-mediated recombination. **(A and B)** 8-week-old *nos>gfp &mCD8gfp* (A) and *nos>gfp & drp1RNAi* mosaic germaria (B) were heat-shocked at 4-weeks-old for 3 days to activate *nos-GAL4*. The germaria with LamC (red, cap cell nuclear envelopes), 1B1 (red, fusomes), ATPase (green, mitochondira) and blue (GFP, the clone cells). A’ and B’ only show ATPase channel. GSCs are outlined by dashed circles. Scale bars, 10 μm.

**Supplementary fig. 8 Defects of mitochondrial redistribution during division and differentiation are observed in *drp1* mutant germ cells. (A-D)** One-week-old *drp1^1^* mutant mosaic germaria with GFP (green, wild-type cells), 1B1 (gray, fusomes) and ATP5ase (red, mitochondria) labeling. Scale bar, 1 μm.
