# Supplementary material for: Aging shifts mitochondrial dynamics toward fission to promote germline stem cell loss
Source: Aging Cell. 2020 Jul 14;19(8):e13191. doi: 10.1111/acel.13191 (PMC7431834; doi:10.1111/acel.13191)
Supplement: Supplementary file 13 — Table S1 [file ACEL-19-e13191-s013.pdf]

Table S1. Mitochondrial dynamics controls GSC maintenance.

Genotypes	clone age	Total germaria	% of Germaria with GSC clone(s)[a]		Fully GSC clone[b]		Partial GSC clone[c]	
FRT19A Ctrl	1W	331	21 ± 2	(100)‡	2 ± 2	(8)π	21 ± 7	(92)π
	2W	293	19 ± 1	(92)	4 ± 4	(20)	14 ± 2	(79)
	3W	336	19 ± 2	(93)	8 ± 2	(36)	14 ± 5	(63)
$Marf^{E}$	1W	362	19 ± 2	(100)‡	2 ± 1	(9)	21 ± 3	(90)
	2W	224	19 ± 5	(93)	3 ± 2	(26)	11 ± 5	(74)
	3W	251	12 ± 3	(60)‖	5 ± 2	(46)	6 ± 3	(53)
$Marf^{B}$	1W	349	20 ± 1	(100)‡	2 ± 1	(7)	22 ± 3	(92)
	2W	296	13 ± 2	(63)‖	3 ± 1	(18)	11 ± 4	(81)
	3W	310	8 ± 2	(38)†	2 ± 2	(15)	6 ± 2	(85)
FRT40A Ctrl	1W	325	20 ± 1	(100)	3 ± 2	(14)π	18 ± 1	(86)π
	2W	306	19 ± 0	(98)	6 ± 3	(31)	13 ± 3	(69)
	3W	331	19 ± 3	(98)	8 ± 2	(39)	13 ± 3	(61)
$Drp1^{2}$	1W	319	20 ± 1	(100)	5 ± 1	(25)*	15 ± 2	(75)*
	2W	227	18 ± 2	(95)	8 ± 2	(44)*	10 ± 1	(56)*
	3W	290	20 ± 1	(106)	10 ± 4	(66)*	6 ± 4	(34)*
$Drp1^{1}$	1W	576	19 ± 4	(100)	9 ± 3	(26)†	26 ± 5	(74)†
	2W	628	15 ± 4	(89)	21 ± 4	(63)†	13 ± 6	(37)†
	3W	571	17 ± 4	(89)	29 ± 5	(92)†	3 ± 3	(8)†
mCD8gfp ctrl	5W	119	79	(100)	43	(54)π	36	(46)π
	8W	117	54	(69.5)	28	(52)	26	(48)
Drp1 KD	5W	106	69	(100)	28	(41)π	41	(59)
	8W	95	63	(102)	51	(81)‖	12	(19)‖

a, Germaria carrying at least one clonally-marked GSC;

b, Germaria carrying GSCs that are all clonally-marked;

c, Germaria carrying GSCs that are not all clonally-marked.

‡ Percentage of initial (1W) germaria carrying GSC clone(s) remained at 2W and 3W.

∏ Proportion of germaria with fully clone (b) or partial clone (c) in germaria carrying GSC clones (a).

¥ Percentage of clonally-marked GSCs in total GSCs relative to that at 1W.

* Significant difference relative to initial value (1W): *P*<0.05.

‖ Significant difference relative to initial value (1W): *P*<0.01.

† Significant difference relative to initial value (1W): *P*<0.001.

Significant differences shown in the column of average of GSCs per germaria are relative to the controls.
